# Systems Genetic Validation of the SNP-Metabolite Association in Rice Via Metabolite-Pathway-Based Phenome-Wide Association Scans

**DOI:** 10.3389/fpls.2015.01027

**Published:** 2015-11-27

**Authors:** Yaping Lu, Yemao Liu, Xiaohui Niu, Qingyong Yang, Xuehai Hu, Hong-Yu Zhang, Jingbo Xia

**Affiliations:** Hubei Key Laboratory of Agricultural Bioinformatics, College of Informatics, Huazhong Agricultural UniversityWuhan, China

**Keywords:** association scan, upper tier metabolite pathway, Wilcoxon rank-sum test, metabolite, replication

## Abstract

In the post-GWAS (Genome-Wide Association Scan) era, the interpretation of GWAS results is crucial to screen for highly relevant phenotype-genotype association pairs. Based on the single genotype-phenotype association test and a pathway enrichment analysis, we propose a Metabolite-pathway-based Phenome-Wide Association Scan (M-PheWAS) to analyze the key metabolite-SNP pairs in rice and determine the regulatory relationship by assessing similarities in the changes of enzymes and downstream products in a pathway. Two SNPs, sf0315305925 and sf0315308337, were selected using this approach, and their molecular function and regulatory relationship with Enzyme EC:5.5.1.6 and with flavonoids, a significant downstream regulatory metabolite product, were demonstrated. Moreover, a total of 105 crucial SNPs were screened using M-PheWAS, which may be important for metabolite associations.

## Introduction

Since the publication of the sequencing data of the human genome (Lander et al., 2001; Venter et al., 2001) and rice genome (Yu et al., 2002), a large number of genetics and genomics studies have emerged based on the vast and varied information available from genomic data.

The pioneering GWAS work traces back to 2005, when Klein et al. (2005) reported a whole-genome case-control association study for genes involved in age-related macular degeneration (AMD). They used a correlation analysis to study the relationship between phenotypes and genotypes in a whole-genome scan. Klein et al.'s result sparked the study of GWAS. Two years later, approximately 100 new GWAS studies emerged that proposed associations between SNPs and various traits (Naidoo et al., 2011). In 2014, the number of human GWAS publications increased to at least 1751, and these studies examined 11,912 SNPs (http://www.genome.gov/gwastudies/; Welter et al., 2014). GWAS has also been widely used to explore the complex traits in plants, an effort that has identified millions of SNPs and key genes associated with important agronomical traits, such as the yield component, plant architecture, stress tolerance, disease resistance, and flowering time (Han and Huang, 2013).

Although GWAS has been successfully applied to many species, the interpretation of GWAS findings is impractical because most of the screened vital associations are part of a larger region of correlated variants (Hofker et al., 2014). Not only do strong linkage disequilibria hinder the identification of causal SNP variants (van der Sijde et al., 2014), but the majority of identified SNPs are intergenic or lie within the intronic region of genes. These problems all demonstrate that the underlying regulatory mechanisms are not easily understood by merely calculating associations between genotypes and phenotypes (van der Sijde et al., 2014).

In the post-GWAS era, a large number of new emerging data make the interpretation of previous GWAS results a challenge. New methods such as PheWAS (Denny et al., 2010) and pathway-based analyses have been proposed to alleviate this problem.

The emergence of large bodies of electronic medical records (EMRs) may help identify gene-disease associations. Denny et al. (2010) proposed a phenome-wide association scan (PheWAS) based on the International Classification of Disease (ICD9) clinic codes. After using Chi square test algorithm, four of the seven known SNP-disease associations reported by Burton (Burton et al., 2007) and Benjamin (Benjamin et al., 2007) were replicated with sufficient power *P*-values based on previous GWAS data. The PheWAS algorithm also identified 19 previously unknown SNP-disease associations, which indicated that PheWAS analysis can be used to investigate SNP-disease associations. Subsequently, Denny et al. (2013) applied the PheWAS paradigm to relate the entire human genome with EMRs. Their result replicated 66% of associations previously identified with GWAS and revealed 63 pleiotropic associations. This strategy demonstrated the reliability of the PheWAS paradigm. Later, Carroll et al. (2014) developed an R package to automatically run the PheWAS program.

Although PheWAS is also subject to disadvantages, such as a lack of EMR data and population inconsistencies, which affect the ability to validate findings, PheWAS hopefully offers a method to explain GWAS data (Hebbring, 2014). Moreover, PheWAS may combine GWAS findings with additional molecular data and pathway information, which is crucial to functionally characterize the associations. Specifically, current findings have not elucidated the effect of SNP variants on downstream pathways in the context of disease development (van der Sijde et al., 2014).

Similar to PheWAS, pathway-based analyses can also enhance the GWAS findings. This approach utilizes enrichment analysis to screen interested genes that are related to high-ranking SNPs. Because complex molecular networks and cellular pathways are often involved in disease susceptibility and disease progression, Wang et al. (2007, 2010) relied on prior biological knowledge of genes and pathways to identify targeted genes that are enriched in certain pathways. The enrichment data show that these genes might be involved in pathogenesis as groups.

Because metabolites are of the utmost importance to our understanding of the genetic regulation of biochemical conversion, designing a system-based approach is essential to track the flow of biological information based on the central dogma, i.e., DNA → transcripts → protein → metabolites → phenotypes (Hofker et al., 2014). Several phenotypes have been studied in the field of crop GWAS research, including the yield component, plant architecture, stress tolerance, and disease resistance (Han and Huang, 2013). Among these phenotypes, metabolites are of great concern. Chen et al. (2014) conducted a metabolic genome-wide association study to comprehensively profile 840 metabolites and approximately 6.4 million SNPs. After identifying thousands of key SNPs, they selected 36 candidate genes that may be physiologically and nutritionally important in the regulation of metabolites. Using similar methods for the maize genome, Li et al. (2013) detected 74 genes associated with oil biosynthesis in 1.03 million SNPs.

Based on systems genetics, we herein propose a metabolite-pathway-based Phenome-Wide Association Scan strategy (M-PheWAS) to discover currently unknown relationships in vast amounts of GWAS data. PheWAS analyzes the association of phenotypes (metabolites) and genotypes and uses pathway-based approaches to identify metabolites and enzymes that are enriched in certain pathways. M-PheWAS outputs a smaller group of SNPs than previous GWAS approaches. The molecular mechanism relating genotypes to phenotypes is then interpreted based on systematic omics data. Finally, 273 SNP-target gene pairs referring to 105 unique SNPs were obtained from evidence based on 74 gene-EC pair and 52 unique enzyme matches identified by pathway enrichment.

## Material and methods

### Data set

The population used in this work consisted of 533 diverse accessions of *Oryza sativa*, including 200 varieties from China, 132 lines from the International Rice Molecular Breeding Program, and 148 varieties from the US Department of Agriculture rice gene bank. These datasets primarily consisted of two subspecies of rice: indica and japonica (Chen et al., 2014).

**Figure 1:**
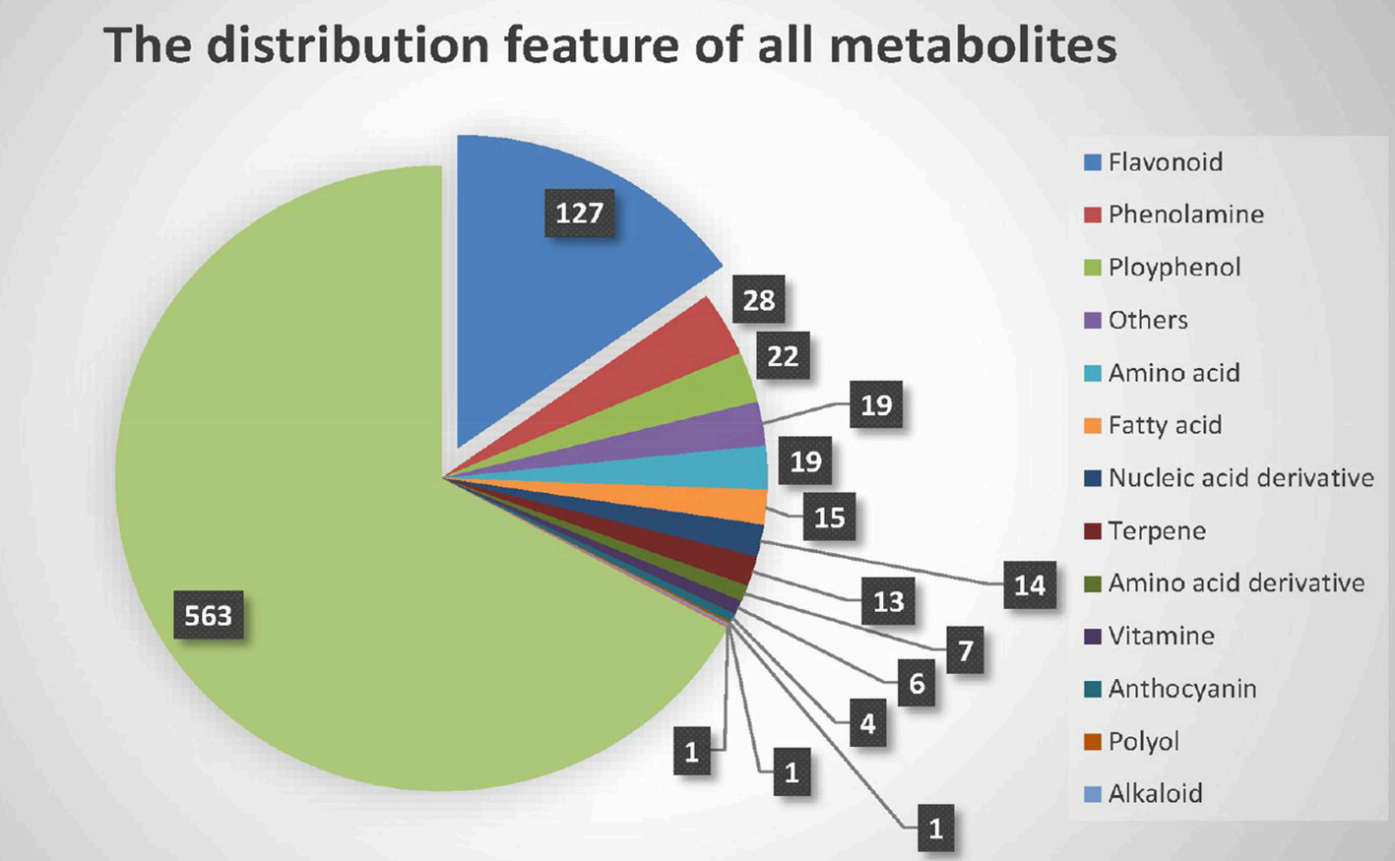
**Class of metabolites**.

Chen et al. built the metabolite dataset (Chen et al., 2014) using liquid chromatography tandem mass spectrometry (LC-MS/MS). In total, 840 distinct metabolic traits in the leaves of rice plants at the five-leaf stage were obtained. Of these metabolites, only 277 were identified or annotated, and 563 remained unknown. Generally, the identified metabolites can be divided into 14 different secondary classes, as shown in Figure 1. Most of the known metabolites are flavonoids, which participate in many vital molecular biological processes, such as UV filtration, symbiotic nitrogen fixation and floral pigmentation (Galeotti et al., 2008).

Metabolites are accumulated in these subspecies in two specific manners. In indica, most metabolites are C-glycosylated and malonylated flavonoids, whereas phenolamides and arabidopyl alcohol derivatives are found in japonica.

The genotype data, which included 6,551,358 high-quality SNPs, were collected from a report by Xie et al. (Xie et al., 2010; Chen et al., 2014). To obtain these SNPs, Chen et al. used BWA 0.6.1 to align the reads to pseudomolecules and then used SAMtools and BCFtools to identify SNPs. Quality control (QC) was conducted to ensure that the minor allele is present at least five times in the population to obtain high-quality SNPs. For indica, japonica and the entire population, Chen et al. used 2,767,191, 1,857,866 and 3,916,415 SNPs in GWAS, respectively (Xie et al., 2010; Chen et al., 2014). These SNPs are all included in our database, in which the minor allele was present at least five times and its frequency was 0.05.

For screened SNPs, the target gene information was obtained from a search for expressed quantitative trait loci within 210 recombinant inbred lines, which detected 13,647 eQTLs for 10,725 e-traits (Wang et al., 2014). The eQTL database consists of probeset, LOD value, target gene name, gene annotation, and cic/trans regulation, and the data are available in Wang's work (Wang et al., 2014; http://jxb.oxfordjournals.org/content/early/2014/01/12/jxb.ert464/suppl/DC1).

### M-PheWAS methodologies

The M-PheWAS strategy evaluates the association of SNP-metabolites based on a non-parametric test. For a fixed metabolite, we separated 533 lines into two categories, A and B, according to different gene types, i.e., SNPs. Subsequently, the Wilcoxon rank-sum test (Wilcoxon, 1945), a nonparametric alternative to the two sample *t*-test, was applied to evaluate the association of a metabolite with a SNP.

The Wilcoxon rank-sum test is based solely on the sum of the order in which the observations from the two samples fall. The *H*_0_ hypothesis holds that the distribution of X-measurements in population *A* is the same as that in *B*,

$$H_{0}:A=B.$$

For *H*_**1**_: *A* > *B*, the *p*-value is

$$p-\text{value}=\text{pr}(W_{A}\geq w_{A}),$$

where *w*_*A*_ denotes the observed rank sum for observations from *A*, and *W*_*A*_ represents the corresponding random variable. The magnitude of the *p*-value inversely correlates with the strength of the association between the SNP and metabolite.

The Bonferroni correction was used to account for multiple tests, and only *p*-values of SNPs that satisfied *p* < 0.05/840 = 5.95 × 10^−5^ were considered significant.

The selected metabolite pathway was then subjected to an enrichment analysis. The metabolites that correspond to a fixed SNP should cluster into a specific class to function consistently. Information on the metabolites related to each SNP was then retrieved and clustered into upper tier class of metabolites. In the corresponding pathway, we attempted to enrich related metabolites in one KEGG pathway based on the classification of the metabolites.

Furthermore, the eQTL data obtained by Wang et al. (2014) were utilized to harvest the SNP-eQTL relationship and the target gene of the SNP-eQTL pair. These target genes were used to obtain the corresponding protein-coding UniProt sequences (probe information for the RNA microarray offered by AffyMetrix), and the EC numbers (Enzyme Commission number) of the protein-coding genes were retrieved from the the UniProt/SwissProt database based on the UniProt codes (http://www.uniprot.org/).

**Figure 2:**
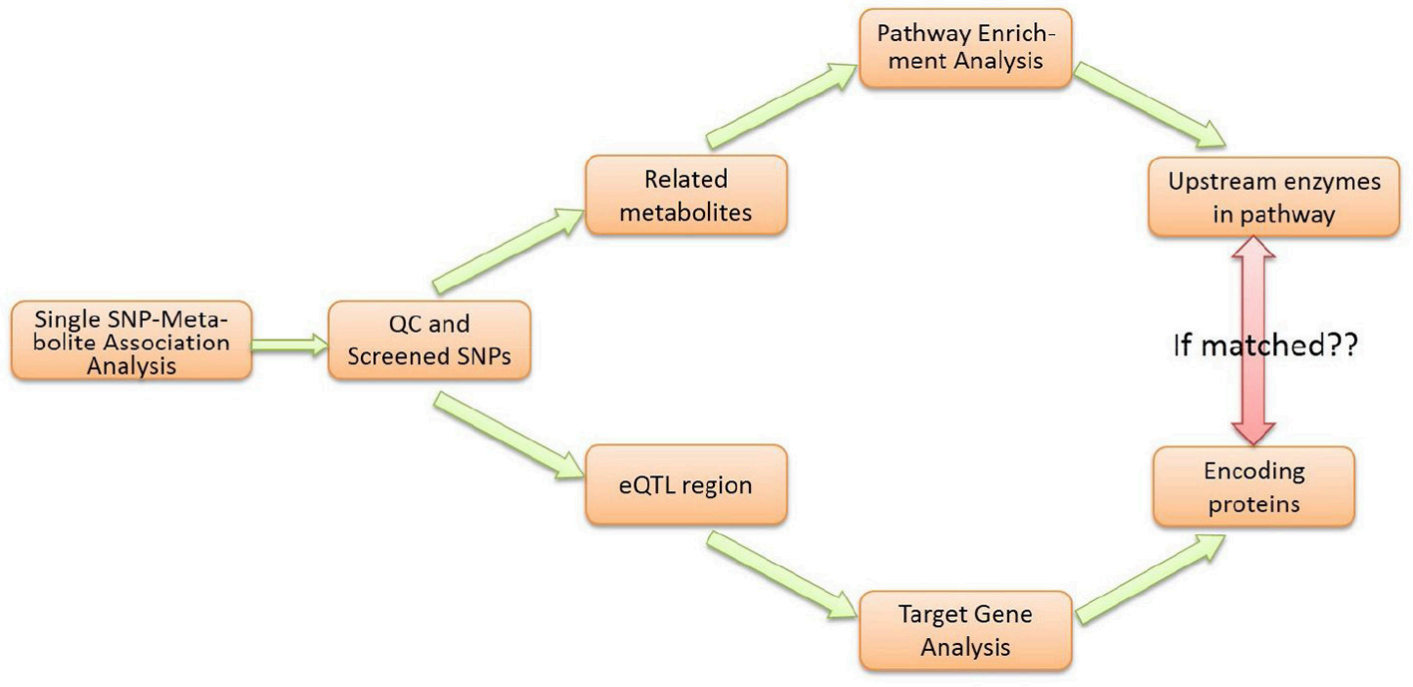
**Flowchart of M-PheWAS strategy**.

As shown in the workflow diagram (Figure 2), the M-PheWAS strategy is based on two parallel molecular biologic processes, i.e., the enrichment analysis of the pathway and the eQTL target gene analysis.

Initially, a Wilcoxon rank sum test (with Bonferroni adjustment) was used to study the genotype-phenotype associations for each genotype (i.e., SNP). This approach yielded a group of strongly associated metabolites for each SNP. For QC, we checked each SNP-metabolite relationship and deleted SNP-metabolite association pairs that contained the unknown metabolites based on a mass spectrum analysis.

Each SNP that passed the single SNP-metabolite analysis and QC was analyzed in parallel using two methods. First, a pathway enrichment analysis was conducted to determine the related Upper Tier Metabolite Pathway (UTMP). Second, eQTL information was used to direct SNPs to target genes and enzymes. The identified UTMP pathway was then searched for targeted enzymes upstream of the target metabolite. The consistency of the enzyme level and downstream metabolite level was checked to verify the effect of the genotype due to SNP variations.

## Results

### Distribution of metabolites

**Figure 3:**
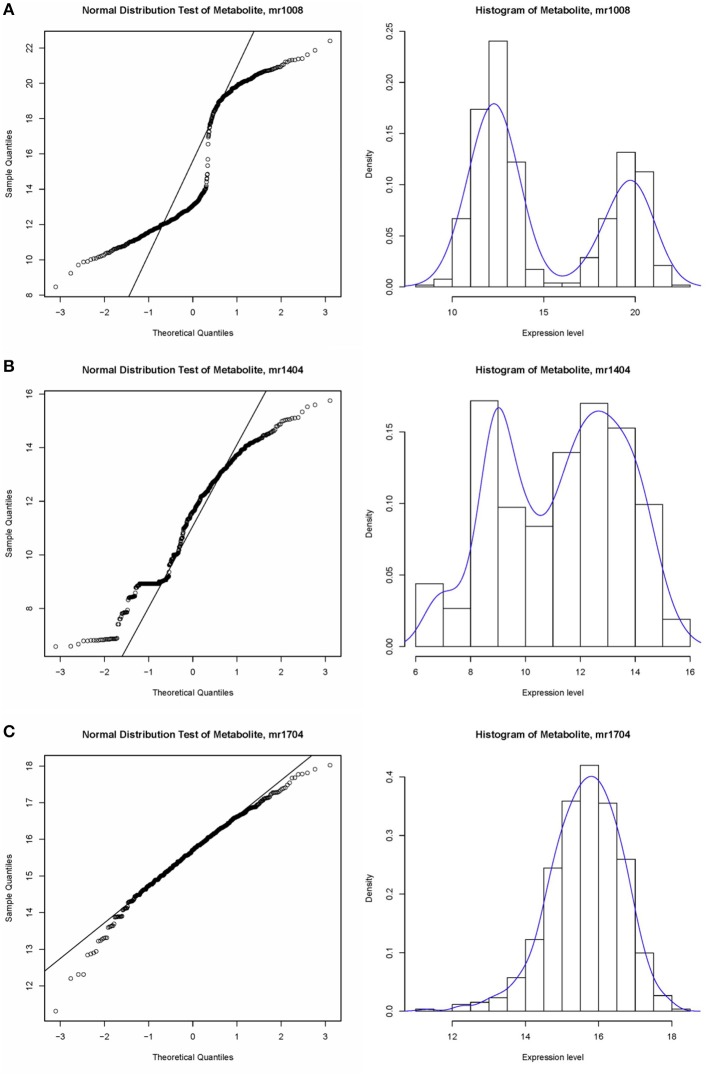
**Example of the data structure of metabolite expression level value. (A)** Normal distribution test of metabolite, sample mr1008, which does not satisfy a normal distribution; **(B)** Histogram of metabolite, sample mr1404, which does not satisfy a normal distribution. **(C)** Histogram of metabolite, sample mr1704, which satisfies the normal distribution.

A non-parametric test is suitable for identifying associations in metabolite expression data. An observation of the data structure did not indicate underlying consistent distributions in expression values. As shown in Figure 3, some of the expression levels satisfied a normal distribution, e.g., mr1704 (unknown), whereas some bar plots showed two peaks, such as that for mr1008 (N-Sinapoylputrescine). In fact, trends in distribution could not be identified for most of metabolites, e.g., mr1404 (unknown). The Wilcoxon test is mainly applied to compare two non-normal distributions or distributions with dissimilar shapes and medians. A Gaussian distribution test was utilized to assess the normality of the metabolite data.

Second, the significance of proportion of flavonoids was analyzed. Because the proportion of flavonoids in the metabolites of the raw data was high, we used a binomial test to confirm that the metabolites of flavonoids identified by M-PheWAS were non-randomly distributed. The raw dataset contained 127 flavonoid compounds of a total of 840 metabolites. Furthermore, seven classes of metabolites are known for the SNP sf0315308337, including four flavonoids identified by M-PheWAS. We used a binomial test to assess the significance, which yielded a *p*-value of 0.01245. Similarly, we predict that the proportion of flavonoid among unknown metabolites is also high.

### SNP-metabolite associations screened by M-PheWAS

**Figure 4:**
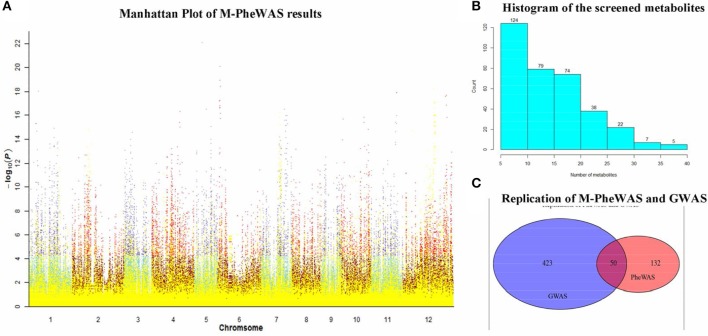
**SNP-metabolite association in rice genome. (A)** is the Manhattan plot of M-PheWAS results. The class-known metabolites are denoted in yellow, whereas the other metabolites are indicated in other colors. The class-unknown metabolites are plotted in two color pairs, sky blue-dark red and slate blue-red, depending on the *p*-value of the metabolite (above or below 5.95 × 10^−5^); The bar plot of metabolite distribution of SNPs is shown in **(B)**; the replication of M-PheWAS and GWAS is shown in **(C)**.

The SNP-metabolite association database from GWAS consists of 2947 SNPs and 840 metabolites (Chen et al., 2014), whereas the distribution of metabolites indicates a non-Gaussian distribution, as shown in Figure S1. Therefore, a non-parametric method, the Wilcoxon rank sum test, was used to evaluate the key associations (at least one SNP to one metabolite). Subsequently, 710 SNPs remained after Bonferroni adjustment. Each SNP corresponded to an average of 20 metabolites. After removing unknown metabolites, the SNP set decreased to 512. To simplify the enrichment strategy, we deleted the SNPs associated with less than five metabolites to yield 282 remaining metabolites. The whole genotype-to-phenotype associations for this dataset are shown (Figure 4A). Furthermore, the Manhattan plots of the M-PheWAS results for each chromosome are shown in Figure S1. Figure 3A shows the distribution of SNPs related to different numbers of metabolites. Specifically, most SNPs were associated with dozens of metabolites. These metabolites were then clustered into corresponding classes. Because the proportion of flavonoids in the metabolite raw data is high, we used a binomial test to confirm that the metabolites of flavonoids identified by M-PheWAS are non-randomly distributed (Figure 4B). The raw dataset contained 127 flavonoids of a total of 840 metabolites. Furthermore, seven metabolite classes are known for the SNP sf0315308337, including four flavonoids identified by M-PheWAS. We used a binomial test to assess the significance, which yielded a *p*-value of 0.01245. Thus, the flavonoid metabolite pathway was then considered the key UTMP pathway, (osa00941, KEGG, http://www.kegg.jp/kegg-bin/show_pathway?osa00941).

To test the repeatability of M-PheWAS and GWAS, we observed the overlap of metabolites from M-PheWAS in GWAS. For SNPs that passed the SNP-metabolite association test in M-PheWAS, we examined the class of corresponding metabolites. For every Metabolite-SNP association, pairs from GWAS and PheWAS were verified when the associated metabolites fell into the same class. M-PheWAS classified 182 metabolites as flavonoids, and 50 of these flavonoids are verified and have 473 metabolites related to flavonoids. These results show that M-PheWAS was able to reliably replicate GWAS SNPs (Figure 4C).

### Validation of the SNP-metabolite associations in systems review

A “general match” strategy was also carried out to supplement the results of the exact match analysis. Specifically, genes coding enzymes involved in flavonoid synthesis (UTMP: osa00941, KEGG) that matched the first three parts of an EC number were defined as a “general match.” Metabolites that clustered in the UTMP pathway clustered into an upper tier class of metabolites. If this class is the same as the enriched class of metabolites from the SNP-metabolite pair, a general match is obtained.

The full results of the general match are shown in Table S2, which provides 105 SNPs and their target gene information.

Exact matches were conducted to search for consistencies in metabolites in a parallel analysis. After implementing the M-PheWAS strategy, an exactly matching metabolite was identified for SNP sf0315305925 and sf0315308337. Specifically, SNP sf0315305925 corresponds to seven metabolites, i.e., Lupulin A, Tricin 4′-O-(syringyl alcohol) ether 5-O-hexoside, Naringenin (in KEGG osa00941), Apigenin 5-O-glucoside, Naringenin O-malonylhexoside, DL-alpha, epsilon-Diaminopimelic acid, and Tricin 4′-O-(B-guaiacylglyceryl) ether 5-O-hexosyl-O-hexoside, whereas SNP sf0315308337 is associated with seven other metabolites, including Lupulin A, Naringenin (in KEGG osa00941), Tricin 4′-O-(syringyl alcohol) ether 5-O-hexoside, DL-alpha epsilon-Diaminopimelic acid, di-C,C-pentosyl-luteolin, Sinapic acid, and a Tricin 4′-O-(syringyl alcohol) ether derivative. These 14 pairs all passed the single SNP-metabolite association scan. Moreover, these metabolites mostly originate from a UTMP flavonoid synthesis process (osa00941, KEGG). Furthermore, this SNP was mapped to the same 40 target genes (Table S1). Among these genes, Q84T92 is the only enzyme of the 40 produced proteins. The UTMP pathway was searched for this enzyme, which indicated that Q84T92 is immediately upstream of the enzyme (EC: 5.5.1.6) of the metabolite Naringenin (C00509, KEGG compound).

### Function analysis and validation of the key metabolites-associated loci

As an exact match, SNP sf0315305925 and sf0315308337 are important SNPs, and these two SNPs share the 40 same target genes. One of these genes encodes the enzyme EC:5.5.1.6, which has been described as “Experimental evidence at transcript level.” This description indicates that the existence of a protein has not been strictly proven but that expression data [such as existence of cDNA(s), RT-PCR or Northern blots] indicate the existence of a transcript (Druka et al., 2003; http://www.uniprot.org/uniprot/Q84T92).

Furthermore, an analysis of metabolite structure shows that metabolites related to these two SNPs correlate. Table 1 shows the corresponding metabolites of screened SNPs, and Table 2 shows the chemical structure of compounds.

**Table 1:** **Corresponding metabolites of screened SNP**.

**SNP sf0315305925**					
**Flavonoid—osa00941**	**EC:5.5.1.6**	**sf0315305925**	**mr1585**	**Lupulin A**	**Terpene**
osa00941	EC:5.5.1.6	sf0315305925	mr1104	Tricin 4′-O-(syringyl alcohol) ether 5-O-hexoside	Flavonoid
osa00941	EC:5.5.1.6	sf0315305925	mr1263	Naringenin(in KEGG osa00941)	Flavonoid
osa00941	EC:5.5.1.6	sf0315305925	mr1437	Apigenin 5-O-glucoside	Flavonoid
osa00941	EC:5.5.1.6	sf0315305925	mr1248	Naringenin O-malonylhexoside	Flavonoid
osa00941	EC:5.5.1.6	sf0315305925	mr1454	DL-alpha, epsilon-Diaminopimelic acid	Others
osa00941	EC:5.5.1.6	sf0315305925	mr1949	Tricin 4′-O-(β-guaiacylglyceryl) ether 5-O-hexosyl-O-hexoside	Flavonoid
**SNP sf0315308337**					
**Flavonoid—osa00941**	**EC:5.5.1.6**	**sf0315308337**	**mr1585**	**Lupulin A**	**Terpene**
osa00941	EC:5.5.1.6	sf0315308337	mr1263	Naringenin(in KEGG osa00941)	Flavonoid
osa00941	EC:5.5.1.6	sf0315308337	mr1104	Tricin 4′-O-(syringyl alcohol) ether 5-O-hexoside	Flavonoid
osa00941	EC:5.5.1.6	sf0315308337	mr1454	DL-alpha, epsilon-Diaminopimelic acid	Others
osa00941	EC:5.5.1.6	sf0315308337	mr1090	di-C,C-pentosyl-luteolin	Flavonoid
osa00941	EC:5.5.1.6	sf0315308337	mr1050	Sinapic acid	Ployphenol
osa00941	EC:5.5.1.6	sf0315308337	mr1206	Tricin 4′-O-(syringyl alcohol)ether derivative	Flavonoid

**Table 2:** **The chemical structure of related metabolites of two screened SNPs**.

**mr1585**	**NA**	
mr1104	C10193	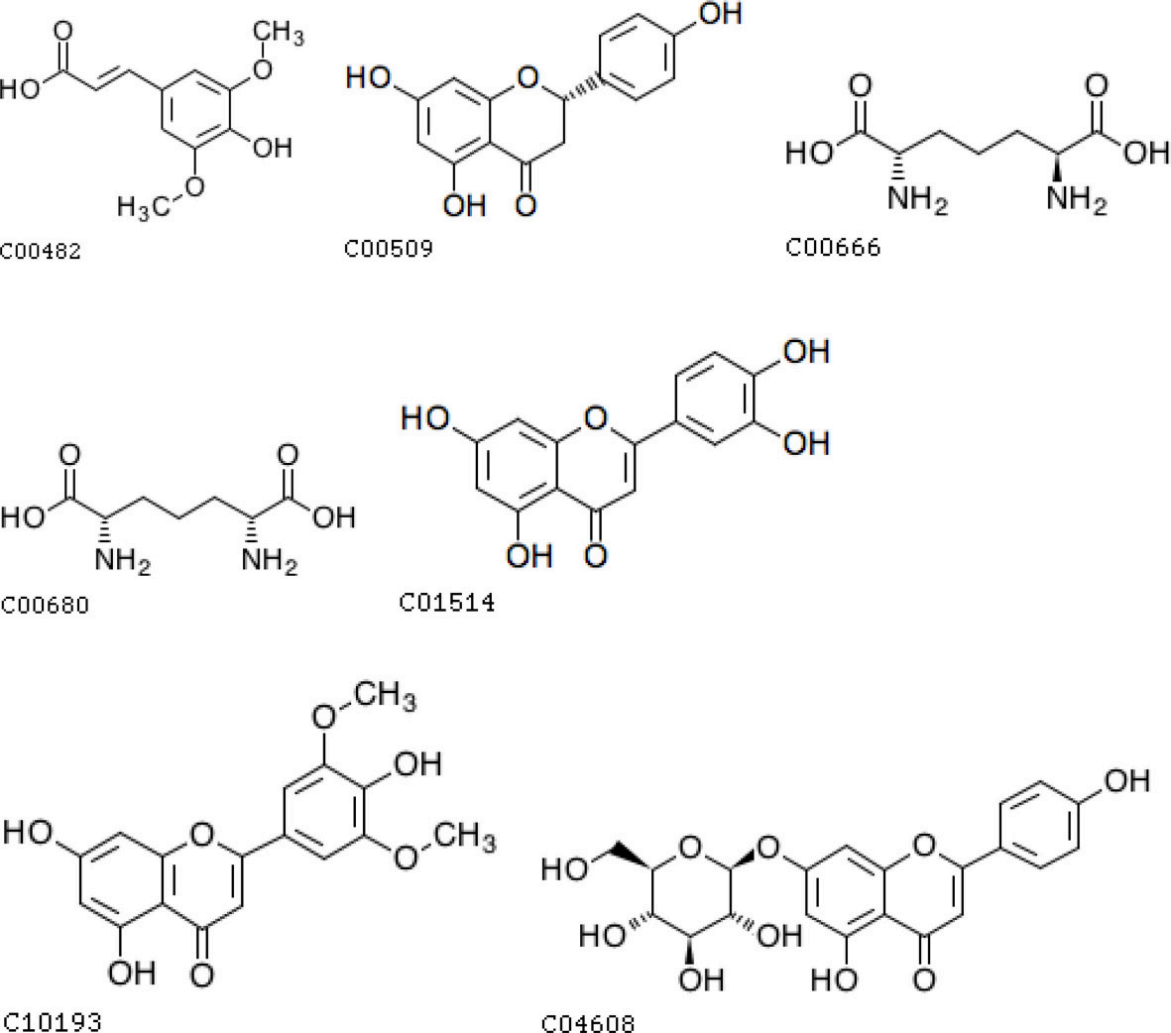
mr1263	C00509	
mr1437	C04608	
mr1248	C00509	
mr1454	C00666, C00680	
mr1949	C10193	
mr1090	C01514	
mr1050	C00482	
mr1206	C10193	

As mentioned above, the two significant SNPs were associated with seven unique known metabolites each, and only 10 unique metabolites were involved in total. Among these 10 metabolites, nine exactly matched or most likely matched chemical structures according to the compound names of the metabolites, and we obtained seven unique chemical structures (see the figures in Table 2) after searching for these metabolites names in KEGG Compound database. The four unique chemical structures (C10193, C00509, C04608, C01514) clearly belong to a flavonoid class and are the derivatives of the flavone. Thus, they all fall into the flavone functional group.

The up-regulation of SNPs and metabolite levels were verified by eQTL data from Wang et al. (2014). Among the 524 lines, 205 SNPs corresponded to a G-A phenotype, which is the same as that in Minghui63, whereas 319 lines corresponded to a C-T genotype. The metabolites differences between the two classes show that a G → C mutation in SNP sf0315305925 and A → T mutation in SNP sf031508337 increase the metabolite levels. The Wilcoxon rank sum test shows a strong significance with a *p*-value of 2.2 × 10^−16^, which proves that the mutation in these two SNPs results in the up-regulation of metabolites. The results of this analysis are shown in Table 3.

**Table 3:** **Significance analysis of up-regulation of SNP toward metabolites level**.

	**SNP sf0315305925**	**Avg metabolite level**	**SNP sf0315308337**	**Avg metabolite level**
Reference Genotype	G		A	
(G, A)-Genotype (Minghui63)	G (205 lines)	9.9935	A (205 lines)	10.0089
(C, T)-Genotype (Zhenshan97)	C (319 lines)	10.5151	T (319 lines)	10.5052

Similarly, the up-regulation of mRNA tended to be significant. We selected 216 chips with microarray data from a set of gene-chip data (Xie et al., 2010). Among the 216 lines, three repeats were obtained from Minghui63, three repeats were obtained from Zhenshan97, and the other 210 repeats were obtained from RILs. The two screened SNPs correspond to the same probes in all chips. For the parent line Minghui63, the chip repeats are GSM1192467, GSM1192468, and GSM1192469, whose mRNA expression values were 10.12, 9.124, and 10.499, respectively. For Zhenshan97, the chip repeats are GSM1192470, GSM1192471, and GSM1192472, with mRNA expression values of 8.41, 6.749, and 8.873, respectively. The result shows that mutated SNPs were strongly up-regulated. We tested this significance in a larger population, i.e., the entire RIL group, and the results of this analysis are shown in Table 4.

**Table 4:** **Significance analysis of up-regulation of SNP toward mRNA expression level**.

	**SNP sf0315305925 and SNP sf0315308337**	**Avg mRNA Level**
Reference Genotype	G and A	
(G, A)-Genotype (Minghui63)	G and A (93 lines)	8.5579
(C, T)-Genotype (Zhenshan97)	C and T (117 lines)	8.7650

As shown above, the Wilcoxon rank sum test indicated strong significance with a *p*-value of 2.2 × 10^−16^. The mutations in SNPs result the up-regulation of metabolites. Because the SNP-regulated enzyme and the downstream metabolite are located in fluctuating areas of the UTMP pathway osa00941, we believe this evidence to indicate the effect of SNPs variations on genotype-phenotype relationships.

**Figure 5:**
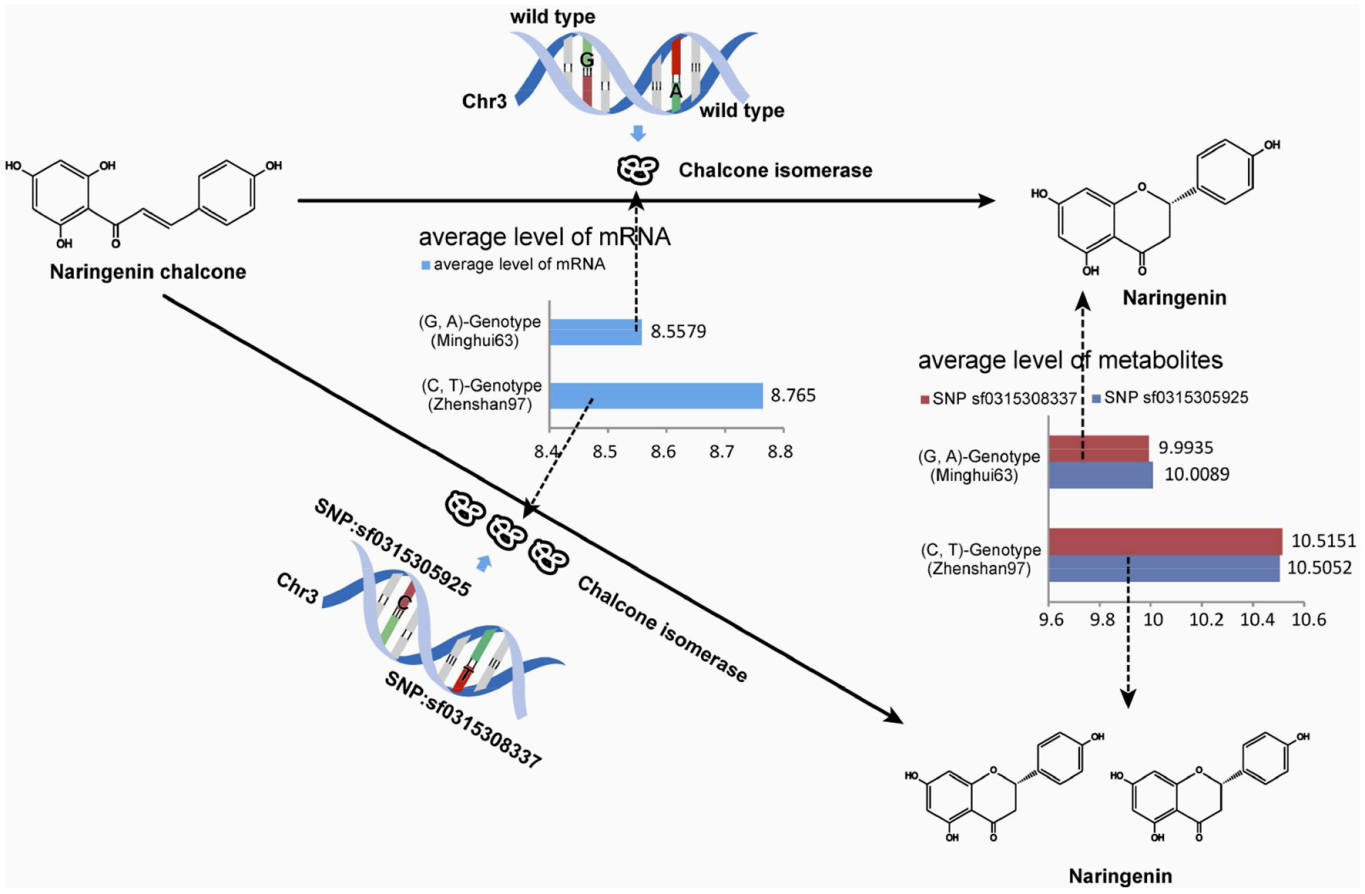
**Up-regulation evidence of SNPs in metabolites pathway**.

Overall, the utility of the M-PheWAS strategy was demonstrated by the consistency of up-regulated of enzymes and downstream products in the UTMP pathway. Specifically, the variations of SNP sf0315305925 and sf0315308337 up-regulate both the enzyme EC: 5.5.1.6 and downstream products, as shown in Figure 5.

## Discussion

GWAS, which is a powerful method for analyzing genotype-phenotype correlations, has been successfully applied to study various species. GWAS has been shown to be essential as an analytical genetic research tool not only for human hereditary diseases and cancer but also for fundamental genetic analyses of other economic species. The advantages of this technique lie in its ability to elucidate the relationship between genetic mutations and a specific phenotype based on the level of network of genetic mutations, i.e., the complex relationships between specific traits and the network of genetic mutations (Hebbring, 2014). Although GWAS has been widely applied to whole-genome research, its coverage unfortunately remains low, which constitutes a significant problem (Hebbring, 2014). PheWAS, which is a relatively new method for association analyses, complements analyses of correlations between mutations and phenotypes (Hebbring et al., 2015), and its ability to identify the known SNP-phenotype associations and predict new SNP-phenotype associations has been validated. Prior to GWAS, PheWAS was developed, which can effectively identify the pleiotropy of a gene and consequently screen out potential phenotypes related to a single mutation. This ability is beneficial to drug research, especially for studies of drug repositioning, side effects and combination. This current hybrid strategy focuses on a combination of PheWAS and EMR data, in which the disease-phenotypes are defined according to codes of the International Classification of Diseases (ICD). However, the definition of phenotypes is highly dependent on the EMR and ICD codes, which limits the applicability of PheWAS—the very problem it was designed to circumvent. Recently, Hebbring used lexical terms obtained from text mining as the phenotypes to strengthen the PheWAS approach (Hebbring et al., 2015).

In this work, we integrated the achievements of previous studies and proposed a typical M-PheWAS strategy. Because the GWAS results are comparatively valuable for reference, we analyzed the published GWAS results (Chen et al., 2014) to sharply reduce the computation time, improve the validation of results, and supplement previous GWAS information. Although PheWAS analyses of diseases or other apparent phenotypes have indicated significant correlations between genotypes and phenotypes, the interpretation of results are far from perfect. The fundamental theory of genetic information transfer indicates the involvement of various inherent processes from genetic mutations to apparent phenotypes, including transcription process from the genome to the transcriptome, translation from the transcriptome to the proteome, the modification, and regulation process from the proteome to metabolome, the regulation and characterization process from the metabolome to the phenome, and epigenetic effects due to the environment that influence the phenome. Because these processes are inherently complex, many unknown factors may affect GWAS and PheWAS. Thus, we herein defined the metabolome of rice as the phenome for PheWAS analysis and then validated the SNP-metabolite associations based on systems genetics to reduce the complexity and uncertainty of information flow from genetic mutations to apparent phenotypes. This approach also allowed us to understand how genetic mutations (or genetic mutation network) act on a specific trait based on the metabolic network.

In this study, we applied a modified PheWAS strategy, M-PheWAS, to perform a customized PheWAS analysis of the published GWAS results and obtain potential SNP-metabolite associations. To test the significance of these association pairs, a Gaussian distribution test was used to assess the distribution of the expression level of each metabolite in different sample lines. These distributions, which are shown in Figure 3, indicate that the majority of metabolites are non-random. Furthermore, the obvious overlap of the PheWAS and GWAS results further indicates that the associations obtained herein are significant. Therefore, the M-PheWAS strategy was proved to be valid. Upon considering the rationality and relative reliability based on systems genetics, we analyzed and identified the biological correlation between genetic mutations and metabolic phenotypes based on various omics data. Although the data available for rice are relatively scarce, our research benefited from the availability of recently obtained omics data. Specifically, we selected flavonoids as metabolites, for which rich information is available, and ultimately identified two “exact matches.” These exact matches demonstrate the rationality and reliability of the M-PheWAS approach and systems genetic validation. Therefore, they shed light on associations that have not yet been identified by systems genetic validation and indicate that these associations can be treated as the predicted SNP-metabolite association pairs, which may serve as a reference for future studies.

Interestingly, visualizing the association pairs by M-PheWAS analysis revealed a cluster of horizontal parallel line segments with the same metabolic points in the post median area of chromosome 12 (Figure S1). This phenomenon indicates that these SNPs correspond to a region of parallel line segments that shares the same regulatory effects, which implies that these SNPs are located in the same regulatory element in the genome. In addition to this phenomenon, similar cases can be found in Figure S1, e.g., the middle and first quarter areas of chromosome 1, the middle and first third areas of chromosome 3, the middle and first third areas of chromosome 6, the last third area of the chromosome 8, and the last third and quarter areas of chromosome 12. The above clusters each represent a regulatory element or signify an interaction among regulatory elements. Overall, we did not observe shared metabolites for different SNPs in different chromosomes. Thus, these clusters are more likely to be regulatory elements rather than interactions of SNPs.

Although the matches that we obtained via M-PheWAS are reliable based on the systems genetics validation, they are not ideal. The number of exact matches was lower than expectation, which constitutes a bottleneck for the analysis of M-PheWAS results. These drawbacks are primarily due to two reasons. First, available data on rice are scarce, and the lack of systematic data affected the systems genetic analysis. Second, the metabolite data and eQTL data used in this research were collected from a mixed sample group, and differences in this sample unpredictably the analysis. Although we generally assumed that most of the underlying genetic regulation and metabolism processes for a given species were the same, the different growing phages may slightly change the physiological status. Thus, a hybrid strategy consists of waiting for more omics data for rice and to develop more efficient algorithms to compensate for this shortage.

In conclusion, our research proved that the improved M-PheWAS efficiently analyzed rice metabolism based on systems genetics. Moreover, we successfully identified and validated important SNP-metabolite association pairs, which provide references for further studies. In addition, the application of PheWAS to study the genetic structure of the metabolome is a novel approach, and we successfully interpreted metabolic phenotypes in a plant. Given the in-depth study of complex traits in plants and the accumulation of data, the application of M-PheWAS to botany will attract increasing attention from researchers.

## Funding

This research is funded by the National Natural Science Foundation of China (Grant no. 61202305) and the Fundamental Research Funds for the Central Universities (Project No. 2013PY120).

### Conflict of interest statement

The authors declare that the research was conducted in the absence of any commercial or financial relationships that could be construed as a potential conflict of interest.
