# Supplementary material for: Systems Genetic Validation of the SNP-Metabolite Association in Rice Via Metabolite-Pathway-Based Phenome-Wide Association Scans
Source: Front Plant Sci. 2015 Nov 27;6:1027. doi: 10.3389/fpls.2015.01027 (PMC4661230; doi:10.3389/fpls.2015.01027)
Supplement: Supplementary file 1 [file Table1.DOC]

**
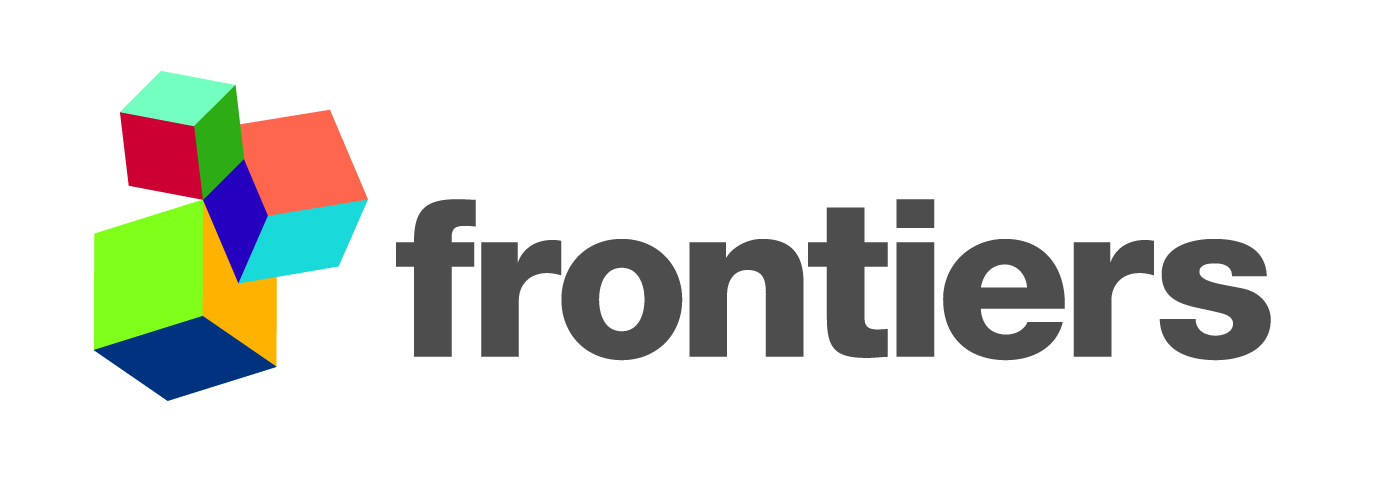
**

***Supplementary Material*:**
**Systems genetic validation of the SNP-metabolite association in rice via metabolite-pathway-based phenome-wide association scans**
**Yaping Lu§, Yemao Liu§, Xiaohui Niu, Qingyong Yang, Xuehai Hu, Hong-Yu Zhang, Jingbo Xia***
*§The same contribution

*Correspondence should be addressed to Jingbo Xia; E-mail: xjb@mail.hzau.edu.cn, [xiajingbo.math@gmail.com](mailto:xiajingbo.math@gmail.com)

**SUPPLEMENTARY TABLES AND FIGURES**

Table S1. List of SNP and regulated gene by eQTL

|  | SNP | Regulated Gene |
| --- | --- | --- |
| 1 | Loc_os07g27810 | Q8W1L6 |
| 2 | Loc_os12g19470 | A6MZQ8 |
| 3 | Loc_os11g03160 | Q336M7 |
| 4 | Loc_os03g25270 | Q10L91 |
| 5 | Loc_os05g30750 | Q60DX8 |
| 6 | Loc_os03g25970 | Q10L91 |
| 7 | Loc_os01g40630 | Q5ZC82 |
| 8 | Loc_os09g36350 | Q69SG5 |
| 9 | Loc_os02g56510 | A2XAI4 |
| 10 | Loc_os03g60509 | Q84T92 |
| 11 | Loc_os04g37500 | B8AUD4 |
| 12 | Loc_os12g38400 | A2XKX4 |
| 13 | Loc_os03g58330 | Q852K0 |
| 14 | Loc_os04g38970 | Q7XUS2 |
| 15 | Loc_os10g23060 | Q339D2 |
| 16 | Loc_os03g27040 | B7E2Y8 |
| 17 | Loc_os01g24710 | Q942L2 |
| 18 | Loc_os03g27010 | B7E2Y8 |
| 19 | Loc_os05g33010 | Q75K78 |
| 20 | Loc_os03g52630 | Q84R49 |
| 21 | Loc_os03g26860 | Q7PC73 |
| 22 | Loc_os03g26970 | B7E2Y8 |
| 23 | Loc_os01g12060 | Q1WM15 |
| 24 | Loc_os04g41100 | Q7XUF4 |
| 25 | Loc_os11g29900 | B8BKF7 |
| 26 | Loc_os07g10920 | Q6ZLK0 |
| 27 | Loc_os01g72870 | Q9LDN2 |
| 28 | Loc_os04g34370 | A2XTE5 |
| 29 | Loc_os03g08220 | Q8H016/Q10QX5 |
| 30 | Loc_os08g40990 | Q84YK7 |
| 31 | Loc_os07g43070 | Q75W16 |
| 32 | Loc_os03g19760 | Q50LH5 |
| 33 | Loc_os09g38768 | B8BE49 |
| 34 | Loc_os01g16340 | Q5NBJ3 |
| 35 | Loc_os03g56270 | Q8W5H8 |
| 36 | Loc_os03g25600 | Q10L91 |
| 37 | Loc_os01g67160 | Q8L4P8 |
| 38 | Loc_os03g26490 | Q7PC73 |
| 39 | Loc_os02g46970 | Q42982 |
| 40 | Loc_os02g17680 | Q6EN45 |
