# Supplementary material for: Systems Genetic Validation of the SNP-Metabolite Association in Rice Via Metabolite-Pathway-Based Phenome-Wide Association Scans
Source: Front Plant Sci. 2015 Nov 27;6:1027. doi: 10.3389/fpls.2015.01027 (PMC4661230; doi:10.3389/fpls.2015.01027)
Supplement: Supplementary file 2 [file Table2.DOC]

**
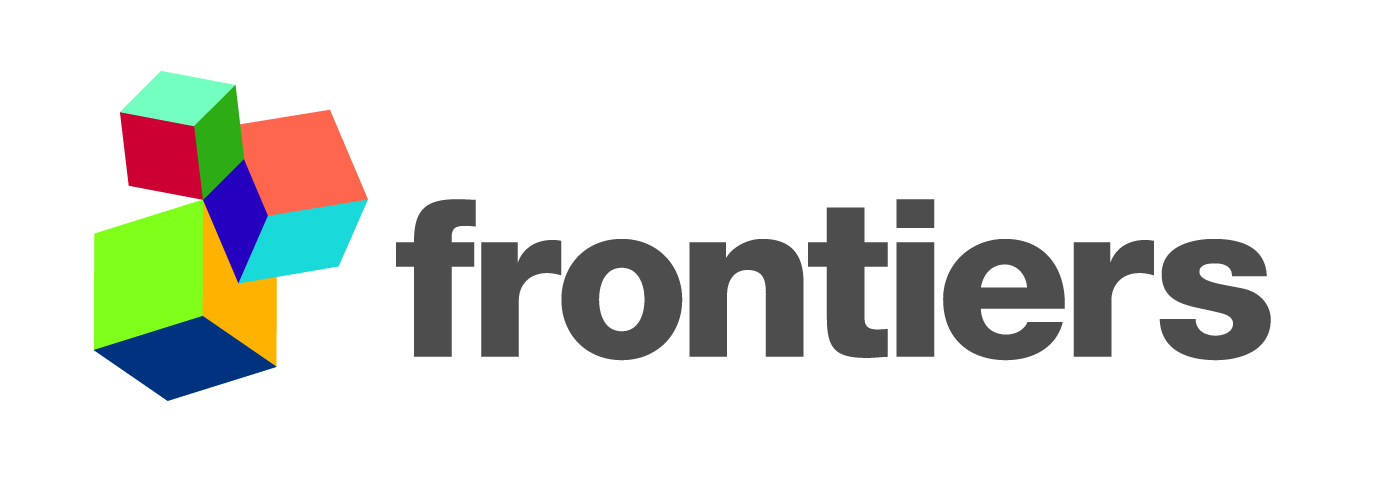
**

***Supplementary Material*:**
**Systems genetic validation of the SNP-metabolite association in rice via metabolite-pathway-based phenome-wide association scans**
**Yaping Lu§, Yemao Liu§, Xiaohui Niu, Qingyong Yang, Xuehai Hu, Hong-Yu Zhang, Jingbo Xia***
*§The same contribution

*Correspondence should be addressed to Jingbo Xia; E-mail: xjb@mail.hzau.edu.cn, [xiajingbo.math@gmail.com](mailto:xiajingbo.math@gmail.com)

**SUPPLEMENTARY TABLES AND FIGURES**

**Table S2. Key SNPs screened by M-PheWAS strategy**

**(The first collumn contains the SNP name, followed with p value for significance of SNP-Metabolite association, target genes through the eQTL, produced enzyme.)**

| **SNP** | | **Average *p* value from PheWAS** | **Target gene (from eQTL)** | **EC number of general match** | **EC number of exact match** |
| --- | --- | --- | --- | --- | --- |
| **sf0100542265** | | 2.16E-05 | LOC_Os06g12460 | EC:2.4.1.- |  |
| **sf0106676498** | | 8.00E-06 | LOC_Os10g37330 | EC:1.1.1.21 |  |
|  |  |  | LOC_Os05g49900 | EC:2.3.1.- |  |
|  |  |  | LOC_Os03g09250 | EC:5.5.1.4 |  |
|  |  |  | LOC_Os07g10600 | EC:2.1.1.41 |  |
|  |  |  | LOC_Os02g38840 | EC:1.1.1.49 |  |
|  |  |  | LOC_Os06g02144 | EC:1.1.1.44 |  |
|  |  |  | LOC_Os04g42920 | EC:1.1.1.42 |  |
|  |  |  | LOC_Os03g17120 | EC:2.3.1.35/EC:2.3.1.1 |  |
|  |  |  | LOC_Os08g38900 | EC:2.1.1.175 |  |
|  |  |  | LOC_Os09g19560 | EC:2.1.1.- |  |
|  |  |  | LOC_Os02g49332 | EC:2.4.1.- |  |
|  |  |  | LOC_Os03g10050 | EC:2.3.1.30 |  |
| **sf0106679441** | | 8.00E-06 | LOC_Os10g37330 | EC:1.1.1.21 |  |
|  |  |  | LOC_Os05g49900 | EC:2.3.1.- |  |
|  |  |  | LOC_Os03g09250 | EC:5.5.1.4 |  |
|  |  |  | LOC_Os07g10600 | EC:2.1.1.41 |  |
|  |  |  | LOC_Os02g38840 | EC:1.1.1.49 |  |
|  |  |  | LOC_Os06g02144 | EC:1.1.1.44 |  |
|  |  |  | LOC_Os04g42920 | EC:1.1.1.42 |  |
|  |  |  | LOC_Os03g17120 | EC:2.3.1.35/EC:2.3.1.1 |  |
|  |  |  | LOC_Os08g38900 | EC:2.1.1.175 |  |
|  |  |  | LOC_Os09g19560 | EC:2.1.1.- |  |
|  |  |  | LOC_Os02g49332 | EC:2.4.1.- |  |
|  |  |  | LOC_Os03g10050 | EC:2.3.1.30 |  |
| **sf0106687727** | | 8.00E-06 | LOC_Os10g37330 | EC:1.1.1.21 |  |
|  |  |  | LOC_Os05g49900 | EC:2.3.1.- |  |
|  |  |  | LOC_Os03g09250 | EC:5.5.1.4 |  |
|  |  |  | LOC_Os07g10600 | EC:2.1.1.41 |  |
|  |  |  | LOC_Os02g38840 | EC:1.1.1.49 |  |
|  |  |  | LOC_Os06g02144 | EC:1.1.1.44 |  |
|  |  |  | LOC_Os04g42920 | EC:1.1.1.42 |  |
|  |  |  | LOC_Os03g17120 | EC:2.3.1.35/EC:2.3.1.1 |  |
|  |  |  | LOC_Os08g38900 | EC:2.1.1.175 |  |
|  |  |  | LOC_Os09g19560 | EC:2.1.1.- |  |
|  |  |  | LOC_Os02g49332 | EC:2.4.1.- |  |
|  |  |  | LOC_Os03g10050 | EC:2.3.1.30 |  |
| **sf0106692749** | | 8.00E-06 | LOC_Os10g37330 | EC:1.1.1.21 |  |
|  |  |  | LOC_Os05g49900 | EC:2.3.1.- |  |
|  |  |  | LOC_Os03g09250 | EC:5.5.1.4 |  |
|  |  |  | LOC_Os07g10600 | EC:2.1.1.41 |  |
|  |  |  | LOC_Os02g38840 | EC:1.1.1.49 |  |
|  |  |  | LOC_Os06g02144 | EC:1.1.1.44 |  |
|  |  |  | LOC_Os04g42920 | EC:1.1.1.42 |  |
|  |  |  | LOC_Os03g17120 | EC:2.3.1.35/EC:2.3.1.1 |  |
|  |  |  | LOC_Os08g38900 | EC:2.1.1.175 |  |
|  |  |  | LOC_Os09g19560 | EC:2.1.1.- |  |
|  |  |  | LOC_Os02g49332 | EC:2.4.1.- |  |
|  |  |  | LOC_Os03g10050 | EC:2.3.1.30 |  |
| **sf0106730123** | | 1.12E-05 | LOC_Os10g37330 | EC:1.1.1.21 |  |
|  |  |  | LOC_Os05g49900 | EC:2.3.1.- |  |
|  |  |  | LOC_Os03g09250 | EC:5.5.1.4 |  |
|  |  |  | LOC_Os07g10600 | EC:2.1.1.41 |  |
|  |  |  | LOC_Os02g38840 | EC:1.1.1.49 |  |
|  |  |  | LOC_Os06g02144 | EC:1.1.1.44 |  |
|  |  |  | LOC_Os04g42920 | EC:1.1.1.42 |  |
|  |  |  | LOC_Os03g17120 | EC:2.3.1.35/EC:2.3.1.1 |  |
|  |  |  | LOC_Os08g38900 | EC:2.1.1.175 |  |
|  |  |  | LOC_Os09g19560 | EC:2.1.1.- |  |
|  |  |  | LOC_Os02g49332 | EC:2.4.1.- |  |
|  |  |  | LOC_Os03g10050 | EC:2.3.1.30 |  |
| **sf0106989017** | | 1.17E-05 | LOC_Os10g37330 | EC:1.1.1.21 |  |
|  |  |  | LOC_Os05g49900 | EC:2.3.1.- |  |
|  |  |  | LOC_Os07g10600 | EC:2.1.1.41 |  |
|  |  |  | LOC_Os02g38840 | EC:1.1.1.49 |  |
|  |  |  | LOC_Os06g02144 | EC:1.1.1.44 |  |
|  |  |  | LOC_Os04g42920 | EC:1.1.1.42 |  |
|  |  |  | LOC_Os03g17120 | EC:2.3.1.35/EC:2.3.1.1 |  |
|  |  |  | LOC_Os08g38900 | EC:2.1.1.175 |  |
|  |  |  | LOC_Os09g19560 | EC:2.1.1.- |  |
|  |  |  | LOC_Os02g49332 | EC:2.4.1.- |  |
|  |  |  | LOC_Os03g10050 | EC:2.3.1.30 |  |
|  |  |  | LOC_Os08g38910 | EC:2.1.1.175 |  |
|  |  |  | LOC_Os07g43390 | EC:2.4.1.25 |  |
|  |  |  | LOC_Os06g42030 | EC:2.4.1.- |  |
|  | **sf0107046780** | 9.38E-06 | LOC_Os10g37330 | EC:1.1.1.21 |  |
|  |  |  | LOC_Os05g49900 | EC:2.3.1.- |  |
|  |  |  | LOC_Os07g10600 | EC:2.1.1.41 |  |
|  |  |  | LOC_Os02g38840 | EC:1.1.1.49 |  |
|  |  |  | LOC_Os06g02144 | EC:1.1.1.44 |  |
|  |  |  | LOC_Os04g42920 | EC:1.1.1.42 |  |
|  |  |  | LOC_Os03g17120 | EC:2.3.1.35/EC:2.3.1.1 |  |
|  |  |  | LOC_Os08g38900 | EC:2.1.1.175 |  |
|  |  |  | LOC_Os09g19560 | EC:2.1.1.- |  |
|  |  |  | LOC_Os02g49332 | EC:2.4.1.- |  |
|  |  |  | LOC_Os03g10050 | EC:2.3.1.30 |  |
|  |  |  | LOC_Os08g38910 | EC:2.1.1.175 |  |
|  |  |  | LOC_Os07g43390 | EC:2.4.1.25 |  |
|  |  |  | LOC_Os06g42030 | EC:2.4.1.- |  |
|  | **sf0107080316** | 1.54E-05 | LOC_Os10g37330 | EC:1.1.1.21 |  |
|  |  |  | LOC_Os05g49900 | EC:2.3.1.- |  |
|  |  |  | LOC_Os07g10600 | EC:2.1.1.41 |  |
|  |  |  | LOC_Os02g38840 | EC:1.1.1.49 |  |
|  |  |  | LOC_Os06g02144 | EC:1.1.1.44 |  |
|  |  |  | LOC_Os04g42920 | EC:1.1.1.42 |  |
|  |  |  | LOC_Os03g17120 | EC:2.3.1.35/EC:2.3.1.1 |  |
|  |  |  | LOC_Os08g38900 | EC:2.1.1.175 |  |
|  |  |  | LOC_Os09g19560 | EC:2.1.1.- |  |
|  |  |  | LOC_Os02g49332 | EC:2.4.1.- |  |
|  |  |  | LOC_Os03g10050 | EC:2.3.1.30 |  |
|  |  |  | LOC_Os08g38910 | EC:2.1.1.175 |  |
|  |  |  | LOC_Os07g43390 | EC:2.4.1.25 |  |
|  |  |  | LOC_Os06g42030 | EC:2.4.1.- |  |
|  | **sf0109354960** | 1.11E-05 | LOC_Os07g43390 | EC:2.4.1.25 |  |
|  |  |  | LOC_Os06g42030 | EC:2.4.1.- |  |
|  | **sf0111192636** | 1.95E-05 | LOC_Os01g29409 | EC:2.1.1.228 |  |
|  | **sf0114830928** | 1.59E-05 | LOC_Os01g27230 | EC:1.3.1.- |  |
|  |  |  | LOC_Os01g29409 | EC:2.1.1.228 |  |
|  |  |  | LOC_Os01g50030 | EC:2.1.1.103 |  |
|  | **sf0128495415** | 1.30E-05 | LOC_Os02g09490 | EC:1.1.1.195 |  |
|  |  |  | LOC_Os08g06100 | EC:2.1.1.42 |  |
|  | **sf0129847641** | 1.65E-05 | LOC_Os02g09490 | EC:1.1.1.195 |  |
|  |  |  | LOC_Os08g06100 | EC:2.1.1.42 |  |
|  | **sf0129865415** | 1.98E-05 | LOC_Os02g09490 | EC:1.1.1.195 |  |
|  |  |  | LOC_Os08g06100 | EC:2.1.1.42 |  |
|  | **sf0129889973** | 1.75E-05 | LOC_Os02g09490 | EC:1.1.1.195 |  |
|  |  |  | LOC_Os08g06100 | EC:2.1.1.42 |  |
|  | **sf0130043352** | 1.18E-05 | LOC_Os01g52260 | EC:2.3.1.30 |  |
|  | **sf0211188934** | 1.87E-05 | LOC_Os01g58740 | EC:1.1.1.8 |  |
|  |  |  | LOC_Os03g17120 | EC:2.3.1.35/EC:2.3.1.1 |  |
|  | **sf0211277320** | 1.00E-05 | LOC_Os01g58740 | EC:1.1.1.8 |  |
|  |  |  | LOC_Os03g17120 | EC:2.3.1.35/EC:2.3.1.1 |  |
|  | **sf0211588650** | 1.91E-05 | LOC_Os01g58740 | EC:1.1.1.8 |  |
|  |  |  | LOC_Os03g17120 | EC:2.3.1.35/EC:2.3.1.1 |  |
|  | **sf0215658517** | 1.09E-05 | LOC_Os03g17120 | EC:2.3.1.35/EC:2.3.1.1 |  |
|  | **sf0216531675** | 8.83E-06 | LOC_Os03g17120 | EC:2.3.1.35/EC:2.3.1.1 |  |
|  | **sf0216621926** | 3.95E-06 | LOC_Os03g17120 | EC:2.3.1.35/EC:2.3.1.1 |  |
|  | **sf0216728688** | 4.59E-06 | LOC_Os03g17120 | EC:2.3.1.35/EC:2.3.1.1 |  |
|  | **sf0216739443** | 8.69E-06 | LOC_Os03g17120 | EC:2.3.1.35/EC:2.3.1.1 |  |
|  | **sf0216741108** | 1.05E-05 | LOC_Os03g17120 | EC:2.3.1.35/EC:2.3.1.1 |  |
|  | **sf0216836178** | 8.25E-06 | LOC_Os03g17120 | EC:2.3.1.35/EC:2.3.1.1 |  |
|  | **sf0216886044** | 1.43E-05 | LOC_Os03g17120 | EC:2.3.1.35/EC:2.3.1.1 |  |
|  | **sf0217832989** | 1.47E-05 | LOC_Os03g17120 | EC:2.3.1.35/EC:2.3.1.1 |  |
|  | **sf0217935126** | 1.63E-05 | LOC_Os03g17120 | EC:2.3.1.35/EC:2.3.1.1 |  |
|  | **sf0233117210** | 1.88E-05 | LOC_Os06g37224 | EC:1.14.13.- |  |
|  | **sf0235317720** | 2.00E-05 | LOC_Os02g57370 | EC:2.3.1.225 |  |
|  |  |  | LOC_Os03g26044 | EC:2.4.1.- |  |
|  |  |  | LOC_Os02g01440 | EC:1.14.13.39 |  |
|  | **sf0306891283** | 1.30E-05 | LOC_Os04g40840 | EC:2.3.1.48 |  |
|  |  |  | LOC_Os04g02730 | EC:1.3.1.- |  |
|  | **sf0307761541** | 1.76E-05 | LOC_Os04g40840 | EC:2.3.1.48 |  |
|  |  |  | LOC_Os04g02730 | EC:1.3.1.- |  |
|  | **sf0309189217** | 1.99E-05 | LOC_Os01g46380 | EC:1.1.1.86 |  |
|  |  |  | LOC_Os01g29409 | EC:2.1.1.228 |  |
|  |  |  | LOC_Os04g40840 | EC:2.3.1.48 |  |
|  |  |  | LOC_Os03g45320 | EC:1.1.1.85 |  |
|  |  |  | LOC_Os09g10600 | EC:1.3.1.9 |  |
|  |  |  | LOC_Os02g24020 | EC:1.17.1.8 |  |
|  |  |  | LOC_Os04g02730 | EC:1.3.1.- |  |
|  |  |  | LOC_Os06g09450 | EC:2.4.1.13 |  |
|  | **sf0311188036** | 1.49E-05 | LOC_Os03g56800 | EC:1.1.1.205 |  |
|  | **sf0311606987** | 1.86E-05 | LOC_Os03g22120 | EC:2.4.1.13 |  |
|  | **sf0311661577** | 1.35E-05 | LOC_Os03g22120 | EC:2.4.1.13 |  |
|  | **sf0315305925** | 1.97E-05 | LOC_Os03g60509 | EC:5.5.1.6 | EC:5.5.1.6 |
|  | **sf0315308337** | 2.29E-05 | LOC_Os03g60509 | EC:5.5.1.6 | EC:5.5.1.6 |
|  | **sf0315711772** | 1.88E-05 | LOC_Os03g26044 | EC:2.4.1.- |  |
|  |  |  | LOC_Os01g50030 | EC:2.1.1.103 |  |
|  |  |  | LOC_Os03g28330 | EC:2.4.1.13 |  |
|  | **sf0317459703** | 1.28E-05 | LOC_Os03g28330 | EC:2.4.1.13 |  |
|  |  |  | LOC_Os10g37330 | EC:1.1.1.21 |  |
|  | **sf0329554165** | 1.71E-05 | LOC_Os05g39650 | EC:1.14.11.18 |  |
|  | **sf0335943790** | 1.13E-05 | LOC_Os03g63970 | EC:1.14.11.- |  |
|  | **sf0400675400** | 1.90E-05 | LOC_Os04g02730 | EC:1.3.1.- |  |
|  | **sf0401697518** | 1.25E-05 | LOC_Os04g02730 | EC:1.3.1.- |  |
|  | **sf0405219091** | 1.28E-05 | LOC_Os04g09654 | EC:2.1.1.- |  |
|  | **sf0428677059** | 1.31E-05 | LOC_Os08g38910 | EC:2.1.1.175 |  |
|  | **sf0434390605** | 1.46E-05 | LOC_Os09g25740 | EC:2.3.1.97 |  |
|  | **sf0521422904** | 7.23E-06 | LOC_Os05g36090 | EC:2.3.1.225 |  |
|  | **sf0604232839** | 2.13E-05 | LOC_Os01g52260 | EC:2.3.1.30 |  |
|  |  |  | LOC_Os04g58200 | EC:1.3.1.33 |  |
|  |  |  | LOC_Os08g33720 | EC:1.1.1.37 |  |
|  |  |  | LOC_Os10g37330 | EC:1.1.1.21 |  |
|  |  |  | LOC_Os05g40260 | EC:2.3.1.- |  |
|  |  |  | LOC_Os07g08200 | EC:2.1.1.295 |  |
|  |  |  | LOC_Os01g46380 | EC:1.1.1.86 |  |
|  | **sf0604335471** | 4.31E-06 | LOC_Os01g52260 | EC:2.3.1.30 |  |
|  |  |  | LOC_Os04g58200 | EC:1.3.1.33 |  |
|  |  |  | LOC_Os08g33720 | EC:1.1.1.37 |  |
|  |  |  | LOC_Os10g37330 | EC:1.1.1.21 |  |
|  |  |  | LOC_Os05g40260 | EC:2.3.1.- |  |
|  |  |  | LOC_Os07g08200 | EC:2.1.1.295 |  |
|  |  |  | LOC_Os01g46380 | EC:1.1.1.86 |  |
|  | **sf0608462868** | 1.69E-05 | LOC_Os01g51170 | EC:2.3.1.97 |  |
|  | **sf0608699391** | 1.05E-05 | LOC_Os01g51170 | EC:2.3.1.97 |  |
|  | **sf0609204987** | 1.61E-05 | LOC_Os01g51170 | EC:2.3.1.97 |  |
|  | **sf0610471974** | 8.03E-06 | LOC_Os05g36090 | EC:2.3.1.225 |  |
|  | **sf0610479622** | 8.03E-06 | LOC_Os05g36090 | EC:2.3.1.225 |  |
|  | **sf0610531673** | 8.03E-06 | LOC_Os05g36090 | EC:2.3.1.225 |  |
|  | **sf0610563320** | 1.65E-05 | LOC_Os05g36090 | EC:2.3.1.225 |  |
|  | **sf0610565085** | 8.03E-06 | LOC_Os05g36090 | EC:2.3.1.225 |  |
|  | **sf0610565238** | 8.03E-06 | LOC_Os05g36090 | EC:2.3.1.225 |  |
|  | **sf0610565344** | 8.03E-06 | LOC_Os05g36090 | EC:2.3.1.225 |  |
|  | **sf0610607096** | 1.37E-05 | LOC_Os05g36090 | EC:2.3.1.225 |  |
|  | **sf0709018381** | 1.55E-05 | LOC_Os03g20300 | EC:1.1.1.49 |  |
|  |  |  | LOC_Os12g42884 | EC:2.1.1.14 |  |
|  |  |  | LOC_Os03g22780 | EC:1.3.1.75 |  |
|  |  |  | LOC_Os02g36210 | EC:5.5.1.13 |  |
|  |  |  | LOC_Os04g09920 | EC:1.14.13.144 |  |
|  |  |  | LOC_Os06g37300 | EC:1.14.13.191/EC:1.14.13.-/EC:1.14.13.78 | |
|  | **sf0725421662** | 1.21E-05 | LOC_Os11g37900 | EC:2.3.1.- |  |
|  | **sf0728264524** | 9.21E-06 | LOC_Os08g38910 | EC:2.1.1.175 |  |
|  |  |  | LOC_Os01g01710 | EC:1.1.1.267 |  |
|  |  |  | LOC_Os07g46790 | EC:2.4.1.25 |  |
|  | **sf0804451576** | 1.14E-05 | LOC_Os08g08200 | EC:2.1.1.56 |  |
|  |  |  | LOC_Os10g20090 | EC:2.4.1.- |  |
|  | **sf0805023492** | 1.40E-05 | LOC_Os08g08200 | EC:2.1.1.56 |  |
|  |  |  | LOC_Os10g20090 | EC:2.4.1.- |  |
|  | **sf0817918355** | 1.90E-05 | LOC_Os02g58480 | EC:2.4.1.13 |  |
|  | **sf0826300504** | 8.63E-06 | LOC_Os06g20400 | EC:2.3.1.225 |  |
|  | **sf0905684207** | 1.25E-05 | LOC_Os09g10600 | EC:1.3.1.9 |  |
|  | **sf0919099461** | 1.30E-05 | LOC_Os11g31640 | EC:2.3.1.50 |  |
|  |  |  | LOC_Os05g49900 | EC:2.3.1.- |  |
|  | **sf0919138866** | 1.61E-05 | LOC_Os11g31640 | EC:2.3.1.50 |  |
|  |  |  | LOC_Os05g49900 | EC:2.3.1.- |  |
|  | **sf0919855123** | 9.76E-06 | LOC_Os05g49900 | EC:2.3.1.- |  |
|  |  |  | LOC_Os02g45110 | EC:2.1.1.62 |  |
|  |  |  | LOC_Os04g40874 | EC:1.1.1.49 |  |
|  | **sf0919877180** | 1.87E-05 | LOC_Os05g49900 | EC:2.3.1.- |  |
|  |  |  | LOC_Os02g45110 | EC:2.1.1.62 |  |
|  |  |  | LOC_Os04g40874 | EC:1.1.1.49 |  |
|  | **sf0920196123** | 1.75E-05 | LOC_Os05g49900 | EC:2.3.1.- |  |
|  |  |  | LOC_Os02g45110 | EC:2.1.1.62 |  |
|  |  |  | LOC_Os04g40874 | EC:1.1.1.49 |  |
|  | **sf0920272026** | 1.81E-05 | LOC_Os05g49900 | EC:2.3.1.- |  |
|  |  |  | LOC_Os02g45110 | EC:2.1.1.62 |  |
|  |  |  | LOC_Os04g40874 | EC:1.1.1.49 |  |
|  | **sf0920348004** | 9.74E-06 | LOC_Os05g49900 | EC:2.3.1.- |  |
|  |  |  | LOC_Os09g34860 | EC:2.3.1.51 |  |
|  |  |  | LOC_Os02g45110 | EC:2.1.1.62 |  |
|  |  |  | LOC_Os08g38910 | EC:2.1.1.175 |  |
|  |  |  | LOC_Os04g40874 | EC:1.1.1.49 |  |
|  | **sf1000078710** | 1.35E-05 | LOC_Os05g08370 | EC:2.4.1.12 |  |
|  | **sf1000442901** | 1.77E-05 | LOC_Os05g08370 | EC:2.4.1.12 |  |
|  | **sf1000575386** | 2.41E-05 | LOC_Os05g08370 | EC:2.4.1.12 |  |
|  | **sf1000902989** | 9.33E-06 | LOC_Os05g08370 | EC:2.4.1.12 |  |
|  | **sf1001437280** | 1.39E-05 | LOC_Os10g02770 | EC:2.4.1.207 |  |
|  | **sf1004607857** | 1.69E-05 | LOC_Os10g11810 | EC:1.1.1.195/EC:2.3.1.30 |  |
|  | **sf1005888770** | 1.40E-05 | LOC_Os10g11810 | EC:1.1.1.195/EC:2.3.1.30 |  |
|  |  |  | LOC_Os10g20090 | EC:2.4.1.- |  |
|  | **sf1006603323** | 1.11E-06 | LOC_Os10g11810 | EC:1.1.1.195/EC:2.3.1.30 |  |
|  |  |  | LOC_Os10g20090 | EC:2.4.1.- |  |
|  | **sf1008135915** | 1.75E-05 | LOC_Os10g11810 | EC:1.1.1.195/EC:2.3.1.30 |  |
|  |  |  | LOC_Os10g20090 | EC:2.4.1.- |  |
|  | **sf1009061971** | 1.35E-05 | LOC_Os10g11810 | EC:1.1.1.195/EC:2.3.1.30 |  |
|  |  |  | LOC_Os10g20090 | EC:2.4.1.- |  |
|  | **sf1009094120** | 1.35E-05 | LOC_Os10g11810 | EC:1.1.1.195/EC:2.3.1.30 |  |
|  |  |  | LOC_Os10g20090 | EC:2.4.1.- |  |
|  | **sf1011051030** | 1.09E-05 | LOC_Os10g11810 | EC:1.1.1.195/EC:2.3.1.30 |  |
|  |  |  | LOC_Os10g20090 | EC:2.4.1.- |  |
|  | **sf1011440598** | 3.77E-06 | LOC_Os10g11810 | EC:1.1.1.195/EC:2.3.1.30 |  |
|  |  |  | LOC_Os10g20090 | EC:2.4.1.- |  |
|  | **sf1011440865** | 1.21E-05 | LOC_Os10g11810 | EC:1.1.1.195/EC:2.3.1.30 |  |
|  |  |  | LOC_Os10g20090 | EC:2.4.1.- |  |
|  | **sf1011580488** | 9.35E-06 | LOC_Os10g20090 | EC:2.4.1.- |  |
|  | **sf1013462182** | 1.51E-05 | LOC_Os10g26630 | EC:2.4.1.- |  |
|  | **sf1022517118** | 1.15E-05 | LOC_Os10g42690 | EC:1.14.11.- |  |
|  | **sf1105540169** | 2.11E-05 | LOC_Os11g10510 | EC:1.1.1.1 |  |
|  | **sf1202864607** | 1.34E-05 | LOC_Os02g58480 | EC:2.4.1.13 |  |
|  | **sf1216525085** | 7.01E-06 | LOC_Os12g29990 | EC:2.3.1.- |  |
|  |  |  | LOC_Os12g29560 | EC:2.3.1.225 |  |
|  | **sf1216552398** | 1.21E-05 | LOC_Os12g29990 | EC:2.3.1.- |  |
|  |  |  | LOC_Os12g29560 | EC:2.3.1.225 |  |
|  | **sf1216924316** | 1.79E-05 | LOC_Os12g29990 | EC:2.3.1.- |  |
|  |  |  | LOC_Os12g29560 | EC:2.3.1.225 |  |
|  | **sf1217047868** | 1.24E-05 | LOC_Os12g29990 | EC:2.3.1.- |  |
|  |  |  | LOC_Os12g29560 | EC:2.3.1.225 |  |
|  | **sf1217251854** | 1.36E-05 | LOC_Os12g29990 | EC:2.3.1.- |  |
|  |  |  | LOC_Os12g29560 | EC:2.3.1.225 |  |
|  | **sf1217320621** | 2.17E-06 | LOC_Os12g29990 | EC:2.3.1.- |  |
|  |  |  | LOC_Os12g29560 | EC:2.3.1.225 |  |
|  | **sf1217321022** | 2.17E-06 | LOC_Os12g29990 | EC:2.3.1.- |  |
|  |  |  | LOC_Os12g29560 | EC:2.3.1.225 |  |
|  | **sf1217324347** | 2.17E-06 | LOC_Os12g29990 | EC:2.3.1.- |  |
|  |  |  | LOC_Os12g29560 | EC:2.3.1.225 |  |
