# Supplementary material for: Systems Genetic Validation of the SNP-Metabolite Association in Rice Via Metabolite-Pathway-Based Phenome-Wide Association Scans
Source: Front Plant Sci. 2015 Nov 27;6:1027. doi: 10.3389/fpls.2015.01027 (PMC4661230; doi:10.3389/fpls.2015.01027)
Supplement: Supplementary file 3 [file Image1.PDF]

Supplementary Material:

Systems genetic validation of the SNP-metabolite association in rice via metabolite-pathway-based phenome-wide association scans

Yaping Lu§, Yemao Liu§, Xiaohui Niu, Qingyong Yang, Xuehai Hu, Hong-Yu Zhang, Jingbo Xia*

* § The same contribution

*Correspondence should be addressed to Jingbo Xia; E-mail: xjb@mail.hzau.edu.cn, xiajingbo.math@gmail.com

SUPPLEMENTARY TABLES AND FIGURES

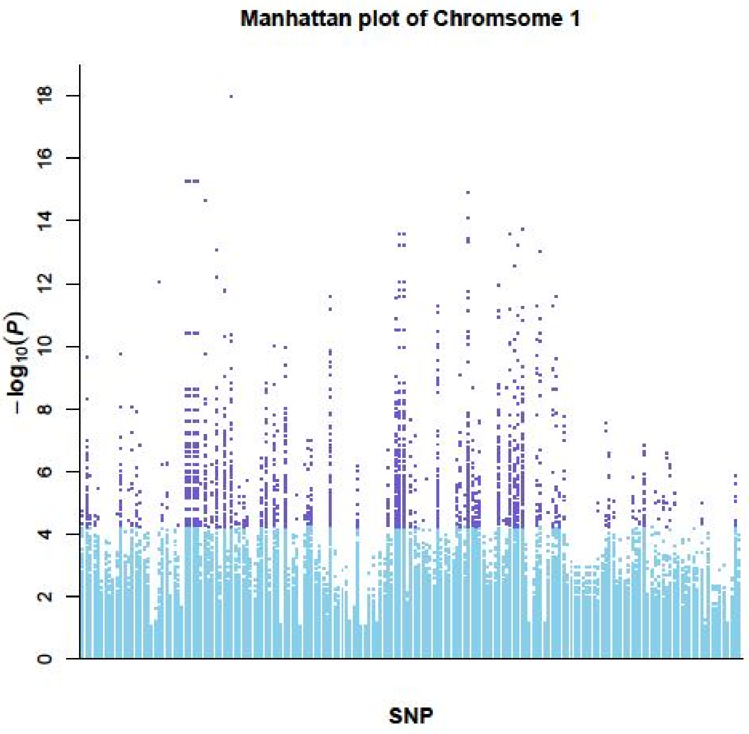

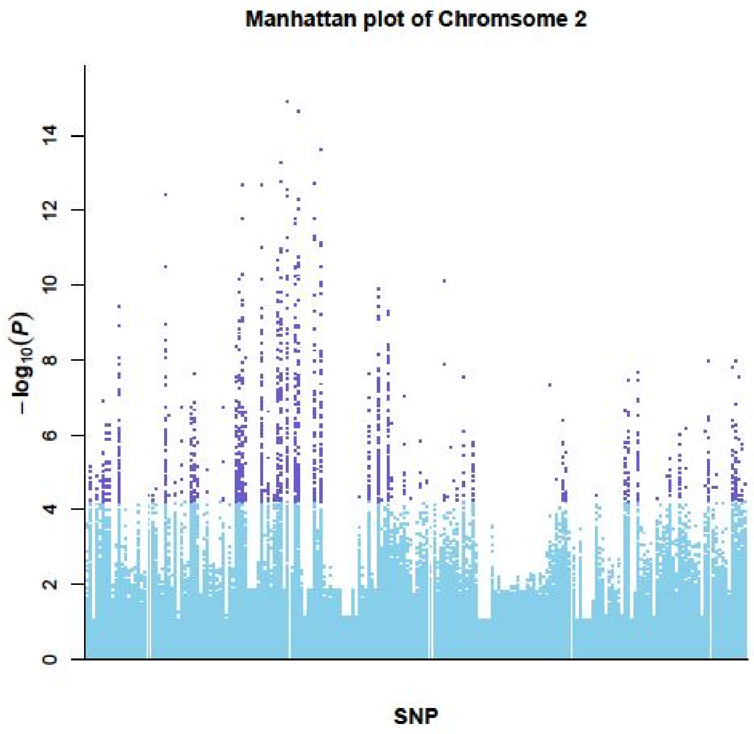
Manhattan plot of Chromsome 2
−log10(P)
0
2
4
6
8
10
12
14
SNP

1

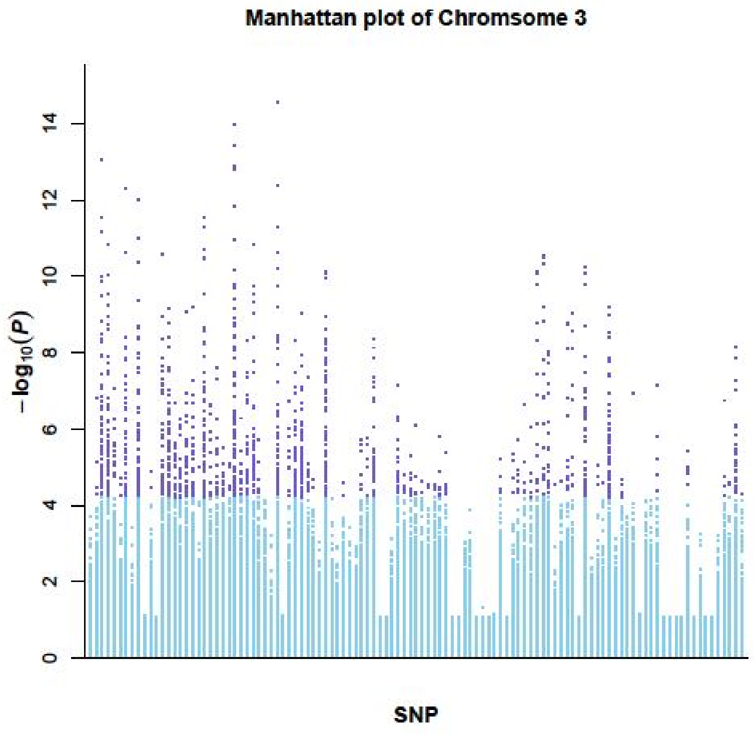
Manhattan plot of Chromsome 3
−log10(P)
0
2
4
6
8
10
12
14
SNP

2

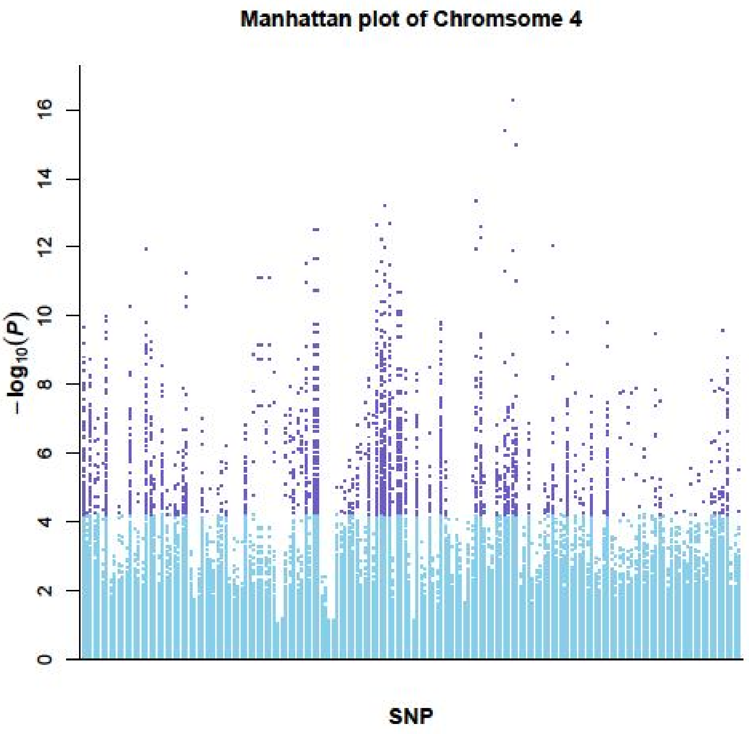
Manhattan plot of Chromsome 4
−log10(P)
0
2
4
6
8
10
12
14
16
SNP

1

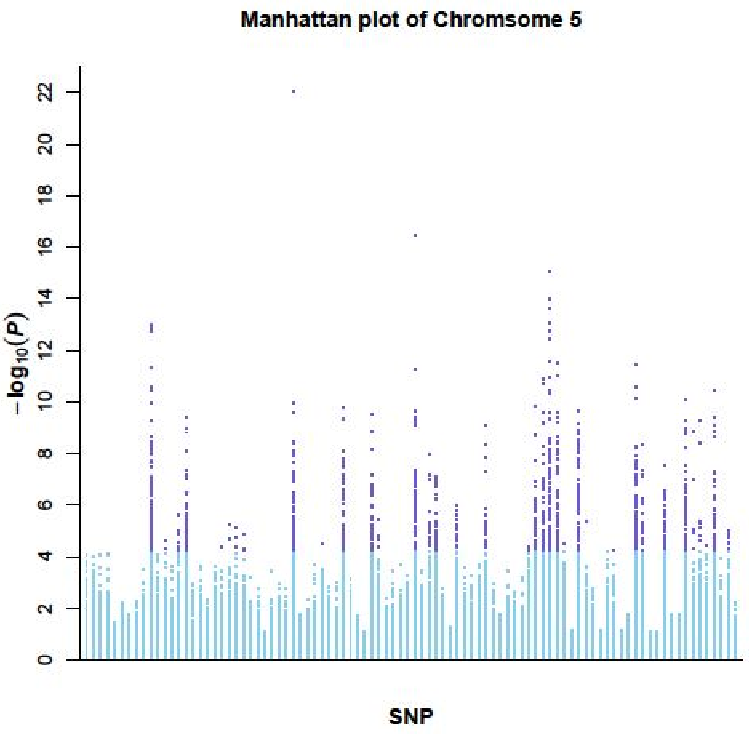
Manhattan plot of Chromsome 5
−log10(P)
0
2
4
6
8
10
12
14
16
18
20
22
SNP

2

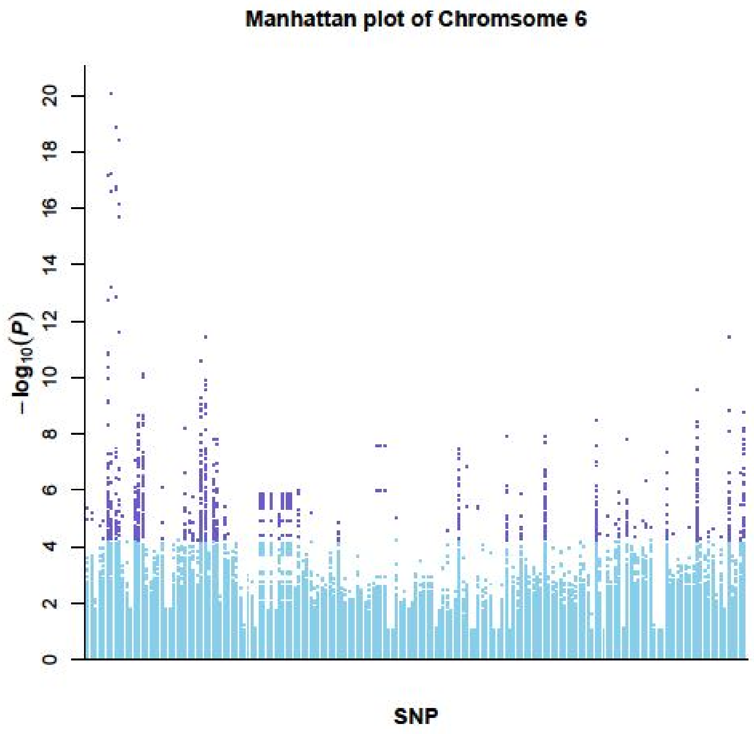
Manhattan plot of Chromsome 6
$-\log_{10}(P)$
0
2
4
6
8
10
12
14
16
18
20
SNP

1

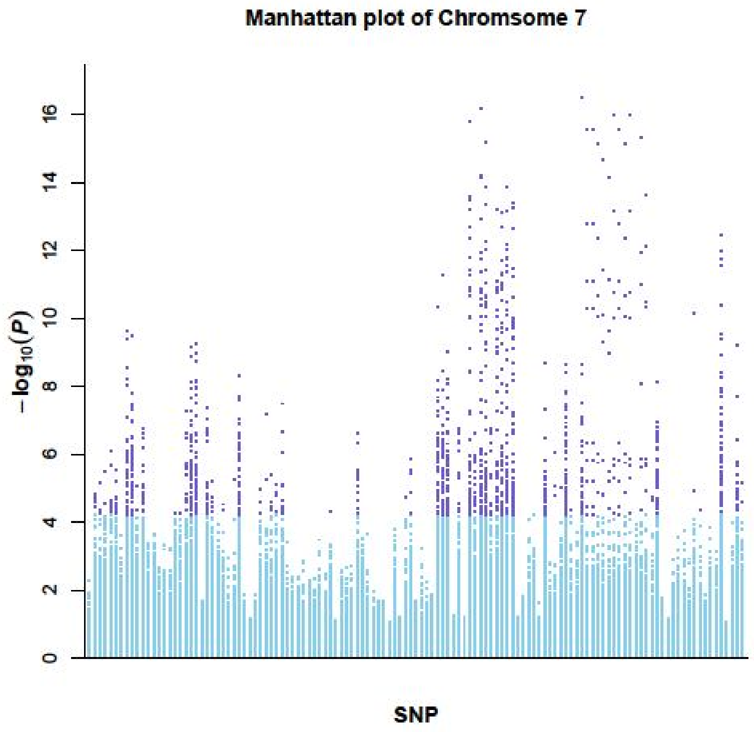
Manhattan plot of Chromsome 7
$-\log_{10}(P)$
0
2
4
6
8
10
12
14
16
SNP

2

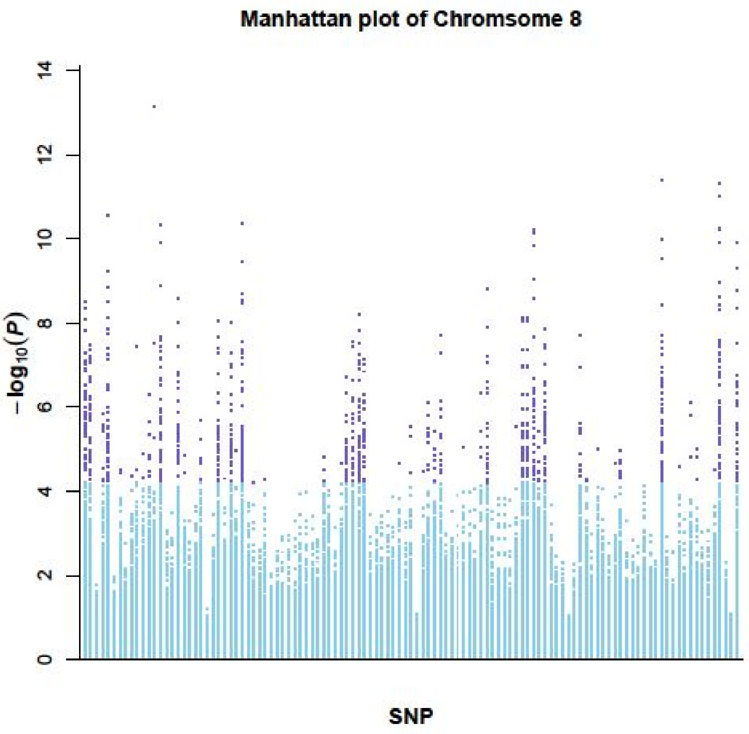
Manhattan plot of Chromsome 8
0
2
4
6
8
10
12
14
−log10(P)
SNP

1

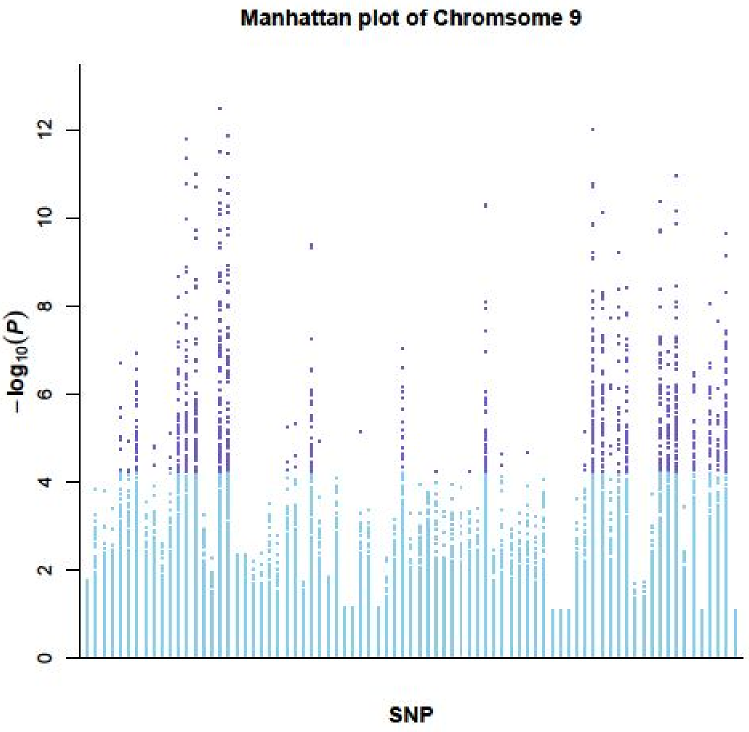
Manhattan plot of Chromsome 9
0
2
4
6
8
10
12
−log10(P)
SNP

2

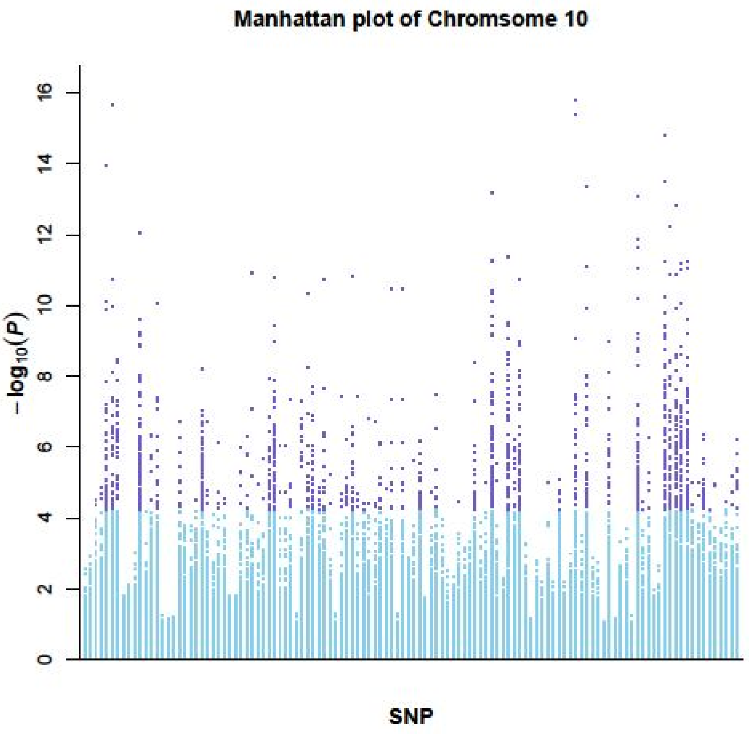
Manhattan plot of Chromsome 10
−log10(P)
0
2
4
6
8
10
12
14
16
SNP

1

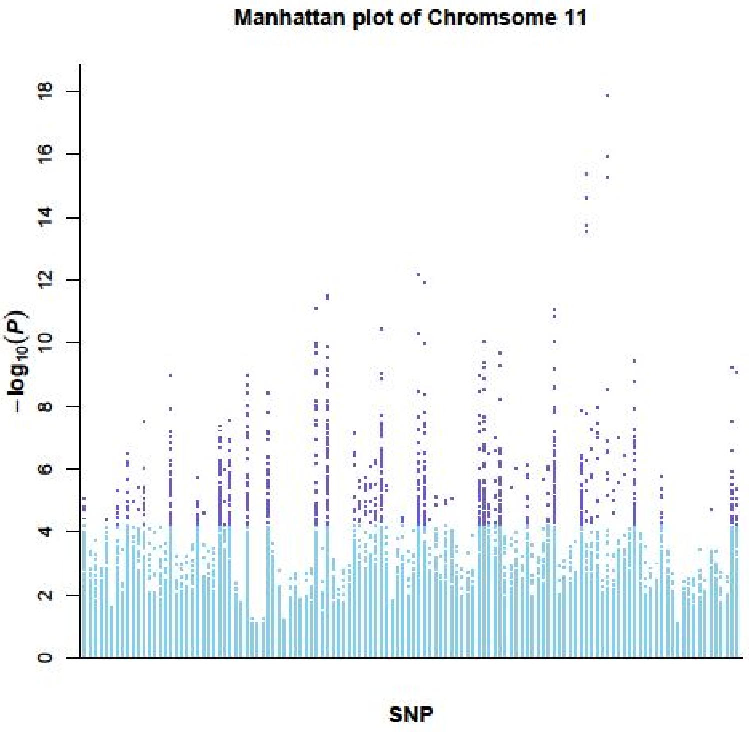
Manhattan plot of Chromsome 11
−log10(P)
0
2
4
6
8
10
12
14
16
18
SNP

2

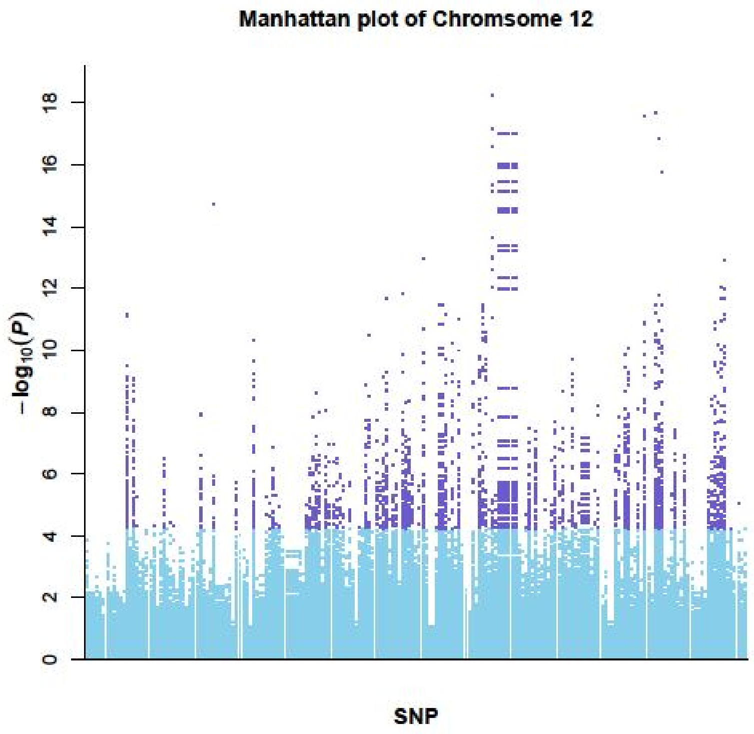

Fig S1. The Manhattan plots of M-PheWAS result for each chromosome
